# Anti-Inflammatory Activities of Yataprasen Thai Traditional Formulary and Its Active Compounds, Beta-Amyrin and Stigmasterol, in RAW264.7 and THP-1 Cells

**DOI:** 10.3390/ph17081018

**Published:** 2024-08-01

**Authors:** Jaenjira Angsusing, Sudarshan Singh, Weerasak Samee, Sarin Tadtong, Leanne Stokes, Maria O’Connell, Hanna Bielecka, Nopparut Toolmal, Supachoke Mangmool, Chuda Chittasupho

**Affiliations:** 1 Ph.D. Degree Program in Pharmacy, Faculty of Pharmacy, Chiang Mai University, CMU Presidential Scholarship, Chiang Mai 50200, Thailand; jaenjira_ang@cmu.ac.th; 2 Thai Traditional Medicine Research Institute, Department of Thai Traditional and Alternative Medicine, Ministry of Public Health, Bangkok 10100, Thailand; nopparut.toolmal@gmail.com; 3 Faculty of Pharmacy, Chiang Mai University, Chiang Mai 50200, Thailand; sudarshansingh83@hotmail.com; 4 Office of Research Administration, Chiang Mai University, Chiang Mai 50200, Thailand; 5 Department of Pharmaceutical Chemistry, Faculty of Pharmacy, Srinakharinwirot University, Nakhon Nayok 26120, Thailand; weerasak@g.swu.ac.th; 6 Department of Pharmacognosy, Faculty of Pharmacy, Srinakharinwirot University, Nakhon Nayok 26120, Thailand; sarin@g.swu.ac.th; 7 School of Pharmacy, University of East Anglia, Norwich, Norwich Research Park, Norfolk NR4 7TJ, UK; l.stokes@uea.ac.uk (L.S.); m.oconnell@uea.ac.uk (M.O.); h.bielecka@uea.ac.uk (H.B.); 8 Department of Pharmaceutical Care, Faculty of Pharmacy, Chiang Mai University, Chiang Mai 50200, Thailand; supachoke.man@cmu.ac.th; 9 Department of Pharmaceutical Sciences, Faculty of Pharmacy, Chiang Mai University, Chiang Mai 50200, Thailand

**Keywords:** natural products, anti-inflammatory activity, antioxidant, Thai traditional medicine formulary

## Abstract

Yataprasen (YTPS) remedy formulary, a national Thai traditional medicine formulary, comprises 13 herbal plants. It has been extensively prescribed to relieve osteoarthritis and musculoskeletal pain in the Thai traditional medicine healthcare system. The aim of this study was to investigate the antioxidant and anti-inflammatory properties of the bioactive compounds (β-amyrin and stigmasterol) of YTPS remedy formulary ethanolic extract, along with its composition. The YTPS formulary extract contains 70.30 nM of β-amyrin and 605.76 nM of stigmasterol. The YTPS formulary extract exhibited ABTS and DPPH free radical scavenging activity, with IC_50_ values of 144.50 ± 2.82 and 31.85 ± 0.18 µg/mL, respectively. The ethanolic extract of YTPS at a concentration of 1000 µg/mL showed a significant (*p* < 0.01) anti-inflammatory effect, mainly by reducing IL-6 and TNF-α release in response to LPS. NO production was prominently lowered by 50% at 24.76 ± 1.48 µg/mL, 55.52 ± 24.40 µM, and more than 570 µM of YTPS formulary extract, β-amyrin, and stigmasterol, respectively. Major components of YTPS, β-amyrin, and stigmasterol exerted significant anti-inflammatory effects by inhibiting LPS-induced IL-1β, IL-6, TNF-α secretion in THP-1 cells. Our findings suggest that the ethanolic extract from YTPS holds promise as an alternative topical treatment for osteoarthritis and inflammatory disorders, potentially with fewer side effects than non-steroidal anti-inflammatory medications (NSAIDs).

## 1. Introduction

Osteoarthritis is one of the most prevalent musculoskeletal conditions among musculoskeletal disorders, affecting almost 343 million worldwide. Mobility and agility are dramatically affected by musculoskeletal diseases, resulting in early retirement, lower quality of life or well-being, and impaired capacity to participate in society [1]. Synovitis is assumed to have a major role in developing pain, joint inflammation, and cartilage breakdown in osteoarthritic joints. Furthermore, nitric oxide (NO) and prostaglandin E_2_ (PGE_2_) have been identified as inflammatory mediators involved in cartilage degradation. Certain pro-inflammatory cytokines, including interleukin-1β (IL-1β), interleukin-6 (IL-6), and tumor necrosis factor (TNF-α), stimulate the expression of cyclooxygenase-2 (COX-2) and nitric oxide synthase (NOS), particularly the inducible isoform of NOS (iNOS). COX-2 and iNOS create significantly high levels of PGE_2_ and NO, respectively, and both play essential roles in inflammation and pain. As a result, local prostanoid concentrations and related pro-inflammatory mediators may be linked to the severity of osteoarthritis [2]. 

Both pharmacological and non-pharmacological treatments are vital approaches to manage patients’ pain and inflammation with knee osteoarthritis; combination therapy is also acceptable. Conventional non-steroidal anti-inflammatory drugs (NSAIDs) have analgesic and anti-inflammatory effects for alleviating mild to moderate pain and inflammatory severity. However, their limitations include the analgesia ceiling effect and several unfavorable adverse effects such as gastrointestinal (GI) side effects, cardiovascular complications, and renal problems. All drug interactions should be considered for the long-term use of NSAIDs. It should be prescribed at the lowest effective dose and for the shortest possible time. Other adverse effects of long-term NSAID medications include impairment of platelet function, prolonged bleeding time, and bronchospasm in some asthma patients after taking aspirin or other NSAIDs [3,4,5,6]. 

Conventional NSAIDs inhibit COX, resulting in reduced PGE_2_ and leading to the vasoconstriction of afferent arterioles and a diminished glomerular filtration rate (GFR). Besides the pharmacodynamic effects of NSAIDs, aging also causes a wide range of pharmacokinetic changes in the elderly, including a decline in volume of distribution, liver function, and renal function compared to young adults. In addition, the elderly tend to encounter more drug interactions than younger patients because they have more chronic diseases or degenerative disorders [7]. The pharmacodynamic effects and pharmacokinetic parameters of NSAIDs must be strictly considered before prescribing in these special populations, along with the warnings to use them with caution. Therefore, medications that can relieve pain and inflammation with minimal side effects have become the preferred treatment for osteoarthritis patients. The treatment may be prescribed as a single treatment or an adjunction with standard treatment such as NSAIDs in order to lower the therapeutic dosage, resulting in fewer adverse events, especially in patients with reduced renal function and elderly.

Interestingly, traditional medicines have also been formulated as topical drugs for the anti-inflammatory disease osteoarthritis. The main pharmacological effects of anti-inflammatory drugs are mainly derived from modulating many inflammatory cytokine levels (e.g., IL-1β, IL-6, TNF-α, PGE_2_), inflammation signaling pathways (e.g., mitogen-activated protein kinases (MAPKs), nuclear factor kappa-light-chain-enhancer of activated B cells (NF-κB)), and inflammatory enzyme (e.g., NOS2, COX-2) downregulation [4]. Plants, namely, *Zingiber officinale* Roscoe, *Artemisia annua* L., *Rosa agrestis* Savi, *Bletilla striata* (Thunb.) Rchb.f., *Boswellia carteri* Birdw., *Salvia miltiorrhiza* Bunge, *Matricaria chamomilla* L., *Tribulus terrestris* L., *Anthriscus sylvestris* (L.) Hoffm., and green tea, have evidence showing their effective use for treating osteoarthritis topically [6].

YTPS remedy formulary, a national Thai traditional medicine formulary, was listed as 58^th^ in “Osot-Pra-Na-Rai”, a traditional Thai medical textbook. The YTPS formulary comprises 13 herbs that have been used to relieve musculoskeletal pain and strain, cramps, and joint pain [8]. It was traditionally formulated in powder dosage form, requiring 40% ethanol or vinegar (naturally fermented) as a vehicle before applying or masking the affected areas 2–3 times per day [8]. However, it was stated that this recipe should not be used for longer than 1 h owing to skin exfoliation; otherwise, it was harmless [8]. 

Among these 13 ingredients in the formulary, *Putranjiva roxburghii*, is the major component of the YTPS formulary. Several chemical compounds have been found in *P. roxburghii,* including β-amyrin and stigmasterol. β-amyrin and stigmasterol are two anti-inflammation markers [9,10] used to identify and quantify of bioactive components in *P. roxburghii* [11]. β-amyrin inhibits COX, LOX, and NOS, leading to decreased release of PGE_2_ and IL-6 [12]. Stigmasterol is one of the plant sterols mostly found in edible plants [13]. Its anti-inflammatory properties help to reduce pro-inflammatory cytokines, including IL-6 and TNF-α [14]. 

While YTPS, a traditional Thai medicinal formulary, has been used for generations, there is limited evidence confirming its anti-inflammatory effect. Moreover, both the ingredients and the formulary itself require pharmacological investigation to assess their potential for anti-inflammatory properties and the underlying mechanism of this effect in THP-1 and RAW264.7 cells. This study aimed to investigate the pharmacological impact of YTPS as an herbal medicine with anti-inflammatory properties.

## 2. Results

### 2.1. Total Phenolic and Flavonoid Content in the YTPS Formulary Extract

The total phenolic content of the YTPS formulary extract was determined by the Folin–Ciocalteu method using the gallic acid standard curve equation, represented as y = 0.01939x + 0.04581, R^2^ = 0.9999. The determination of the flavonoid content was performed through an aluminum chloride colorimetric assay, and the result was calculated using the quercetin standard curve equation: y = 0.001568x + 0.07207, R^2^ = 0.9992. The contents of total phenolic and total flavonoid compounds in the YTPS formulary extract, individual extract, β-amyrin, and stigmasterol were illustrated in Table 1. The standard calibration curve of quercetin and gallic acid can be found in the Supplementary Figure S2.

### 2.2. Identification and Quantification of Bioactive Compounds in the YTPS Formulary Extract Using HPLC Analysis

The HPLC chromatogram demonstrated that the peaks corresponding to both markers aligned with the retention times of the reference standards. The retention times for β-amyrin and stigmasterol were 10.31 and 11.43 min, respectively. The HPLC chromatogram of the YTPS formulary extract spiked with beta-amyrin and stigmasterol can be found in the Supplementary Figure S3. These results suggest that the YTPS formulary extract contained β-amyrin and stigmasterol. The calibration curve of the spiked sample was found to be linear in the range of 5–100 µg/mL, with R^2^ = 0.9952 for β-amyrin and 0.9954 for stigmasterol The standard calibration curve for β-amyrin and stigmasterol using HPLC analysis can be found in Supplementary Figure S4. The YTPS formulary extract contained 0.25 mg of β-amyrin per gram of crude extract and 0.03 mg of stigmasterol per gram of crude extract. 

### 2.3. Anti-Oxidant Activities of the YTPS Formulary Extract

**Figure 1:**
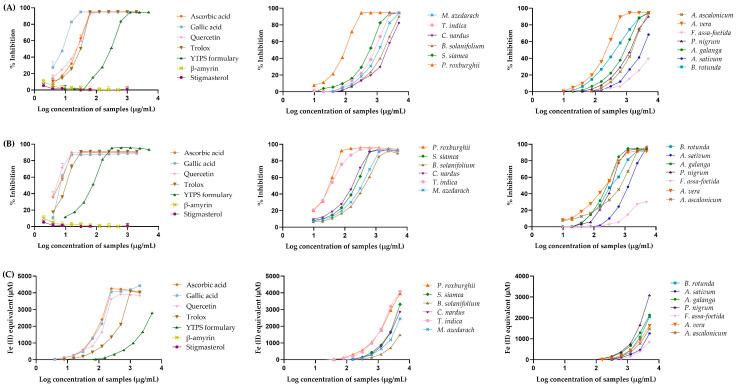
(**A**) ABTS radical scavenging activity; (**B**) DPPH radical scavenging activity; (**C**) FRAP value of ascorbic acid (3.91–2000 µg/mL), gallic acid (3.91–2000 µg/mL), quercetin (3.91–2000 µg/mL), Trolox^®^ (3.91–2000 µg/mL), YTPS formulary extract (9.77–5000 µg/mL), individual extract in the formulary (9.77–5000 µg/mL), β-amyrin (4.57–234 µM), and stigmasterol (4.73–242 µM).

The free radical scavenging activity of the YTPS formulary extract, β-amyrin, stigmasterol and individual extract compared to ascorbic acid, gallic acid, quercetin, and Trolox^®^ were calculated as the %DPPH radical scavenging activity. Ascorbic acid, gallic acid, quercetin, Trolox^®^, and the YTPS formulary, along with all the individual extracts, exhibited dose-dependence for the capacity to scavenge free radicals, as displayed in Figure 1A. The antioxidant activities of the YTPS formulary and individual extracts were confirmed by ABTS free radical scavenging activity and compared with ascorbic acid, gallic acid, quercetin, and Trolox^®^. As shown in Figure 1B, an escalation of %inhibition was observed when the concentrations of these extracts increased. Figure 1C illustrates the ferric-reducing antioxidant capacity of ascorbic acid and YTPS formulary extract. The FRAP value was expressed in terms of Fe^2+^ equivalent. The calibration curve was established from ferrous sulfate (Fe^2+^). The calibration curve was found to be linear across the range of 9.77–2500 µM (R^2^ = 1). The standard curve of ferric reducing antioxidant power assay using ferrous sulfate can be found in Supplementary Figure S5. As the concentration rose, it was noticed that the IC_50_ of the FRAP values of ascorbic acid, gallic acid, quercetin, and the extract of the YTPS formulary increased accordingly. These results indicate that the antioxidant capability of the YTPS formulary extract and its components is related to its ability to reduce ferric ions. The FRAP values of ascorbic acid, gallic acid, quercetin, Trolox^®^, β-amyrin, and stigmasterol were reported at 1000 µg/mL, while the YTPS formulary extract along with the individual extract in the formulary was recorded at 1250 µg/mL. Data are shown in Table 2. However, β-amyrin and stigmasterol did not exhibit antioxidant activity in any antioxidant assays.

### 2.4. In Vitro Cytotoxicity of the YTPS Formulary Extract, β-Amyrin, Stigmasterol, and 11 Individual Extracts 

#### 2.4.1. Cytotoxicity of β-Amyrin, Stigmasterol, and the YTPS Formulary Extract on RAW 264.7 Cells 

**Figure 2:**
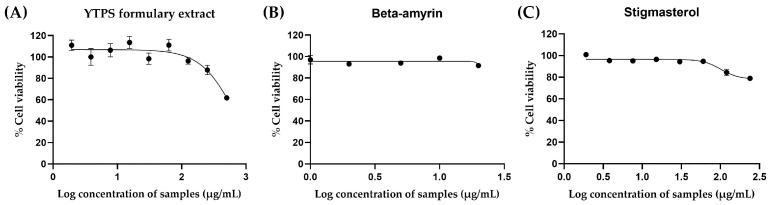
Effect of (**A**) YTPS formulary extract (1–300 µg/mL), (**B**) β-amyrin (1.8–230 µM), and (**C**) stigmasterol (1.88–240 µM) on the cell viability of RAW264.7 cells using MTT assay. Cells were seeded in 96-well plates at a density of 1 × 10^4^ cells/well. The cells were then treated with YTPS formulary extract and incubated for 24 h before conducting the MTT assay. Data are expressed as a percentage of control (media only). Data were gathered from three separate experiments; each was conducted with triplicate wells (n = 3). The descriptive statistics were analyzed by GraphPad Prism version 9.5.0.

The cytotoxicity levels of β-amyrin, stigmasterol, and the YTPS formulary extract against RAW264.7 cells were evaluated to find the maximum concentration able to be used prior to the NO assay, the anti-inflammation test. Cell viability was determined at different concentrations of YTPS formulary extract, ranging from 1.95 to 250 µg/mL, by performing an MTT. The maximum concentration of herbal extract tested was within the range tested by Park et al. [15]. YTPS formulary extract did not cause any cytotoxicity to RAW264.7 cells at tested concentrations after 24 h of incubation. The standard β-amyrin and stigmasterol at varied concentrations between 0.78–100 µg/mL demonstrated no significant toxic effects on RAW264.7 cells. However, at the highest concentration of 100 µg/mL, the cell viability decreased to 71.58 ± 1.45% and 79.72 ± 1.46% after being treated with β-amyrin and stigmasterol compared to the control medium, respectively. The results indicated that the cell viability remained above 80% for YTPS formulary extract at 1–300 µg/mL, β-amyrin at 1.8–230 µM, and stigmasterol at 1.88–240 µM, as depicted in Figure 2. 

#### 2.4.2. Cytotoxicity of β-Amyrin, Stigmasterol, the YTPS Formulary Extract, and 11 Distinct Extracts on THP-1 Cells

**Figure 3:**
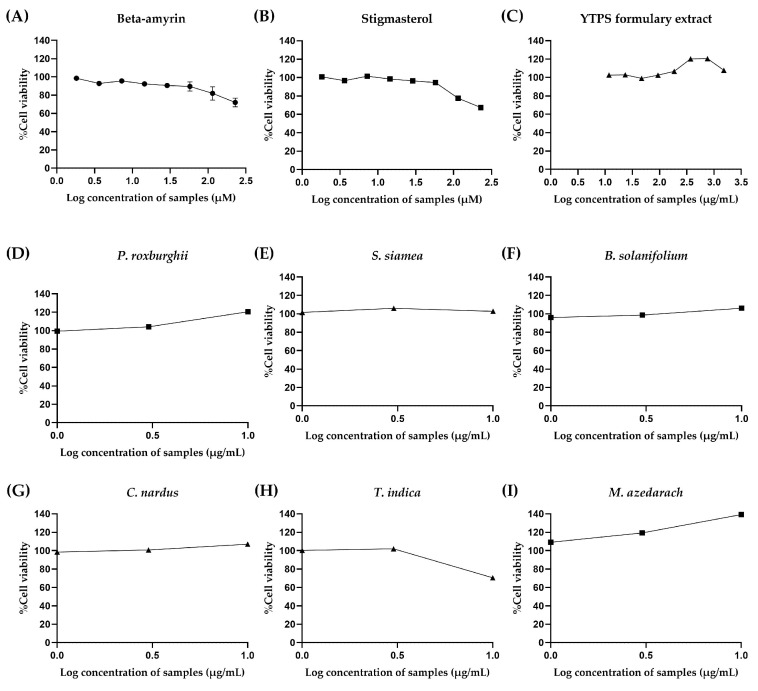

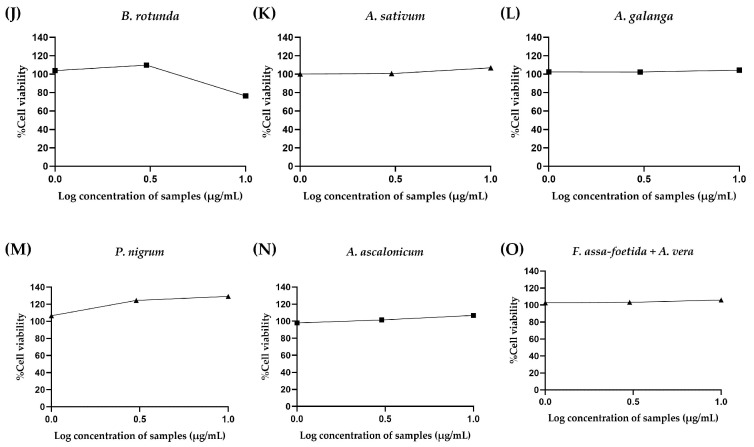
Effect of (**A**) β-amyrin (1.8–230 µM), (**B**) stigmasterol (1.88–240 µM), (**C**) YTPS formulary extract (11.72–1500 µg/mL), and (**D**–**O**) individual extract (10–100 µg/mL) on the cell viability of THP-1 cells. Cell viability was assessed using an Alamar blue metabolic assay by treating the cells with Resazurin dye solution for 4 h after 24 h of incubation of the samples. Alamar blue fluorescence was measured, and data are expressed as a percentage of control (media only). Supernatants were collected and measured with FlexStation^®^ 3 at 570 nm excitation and 600 nm emission using SoftMax Pro 5.4.6 software for data recording and analysis. Data were gathered from three separate experiments, each conducted in triplicate (n = 3). The descriptive statistics were analyzed by GraphPad Prism version 9.5.0.

The THP-1 cells underwent treatment with varying concentrations of the YTPS formulary extract, β-amyrin, stigmasterol, and 11 distinct extracts to determine the acceptable exposure concentrations of samples for measurement of anti-inflammatory and pro-inflammatory cytokines. Neither YTPS nor *P. roxburghii* have been tested on THP-1 cells before. There was an experiment with herbal extract on THP-1 testing which focused on an anti-inflammatory effect using ten times greater than the maximum concentration of 150 µg/mL tested on the same cells [16]. Thus, we increased the concentration to 10 times to find the toxic concentrations of samples on THP-1 cells to test for the anti-inflammation activity at non-toxic concentrations. The results are shown in Figure 3. The concentrations used were within the ranges of 11.72–1500 µg/mL, 1.80–230 µM, 1.88–240 µM, and 10–100 µg/mL, respectively. A decrease in cell viability was observed in THP-1 cells exposed to 120 and 240 µM of stigmasterol, leading to remaining cell viabilities of 67.43 ± 1.93% and 77.52 ± 4.71%, respectively. The THP-1 cells were subjected to treatment with 230 µM of β-amyrin, resulting in a decline in cell viability to 72.02 ± 5.85%. THP-1 cells treated with most of the individual plant ethanolic extracts showed no discernible decrease in cell viability, with the exception of the extracts from the leaves of *T. indica* and the rhizome and roots of *B. rotunda,* which were tested at the maximum concentration of 100 µg/mL, which markedly lowered the cell viability to 70.55 ± 0.56% and 76.30 ± 0.63%, respectively. Interestingly, the findings indicated that ethanolic extract of *P. roxburghii* leaves and *P. nigrum* fruits notably increased the cell viability of THP-1 to 120.5 ± 0.34% and 129.3 ± 3.77% at 100 µg/mL, respectively. 

### 2.5. Anti-Inflammatory Effects of the YTPS Formulary Extract, β-Amyrin, Stigmasterol, and 13 Individual Extracts

#### 2.5.1. Effects of the YTPS Formulary Extract on Nitric Oxide Production

**Figure 4:**
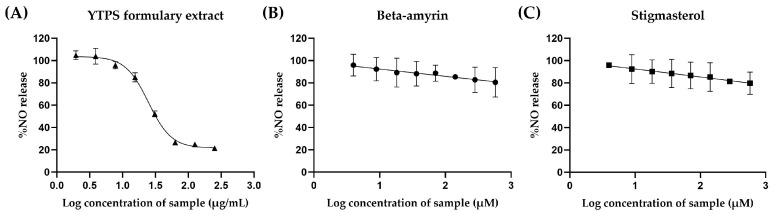
Effects of (**A**) YTPS formulary extract (1.95–250 µg/mL), (**B**) β-amyrin (1.8–230 µM), and (**C**) stigmasterol (1.88–240 µM) on NO release from LPS-treated RAW264.7 cells. Data were gathered from three separate experiments, each conducted in triplicate (n = 3). The data were analyzed by GraphPad Prism version 9.5.0 with one-way ANOVA and Dunnett’s multiple comparisons test (statistical significance denoted by *p* < 0.05 compared with cells treated with LPS).

**Figure 5:**
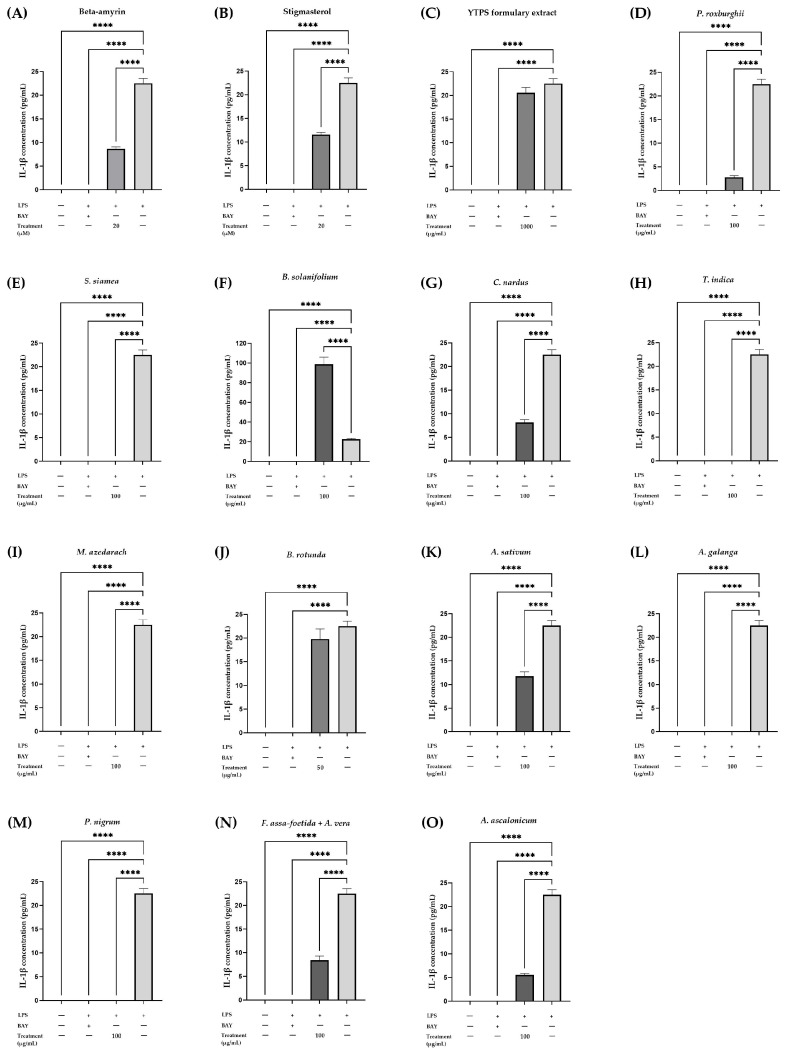
Effects of (**A**) β-amyrin (20 µM), (**B**) stigmasterol (20 µM), (**C**) YTPS formulary extract (1000 µg/mL), and (**D**–**O**) individual extract (50 or 100 µg/mL) tested at non-toxic concentrations on IL-1β release in THP-1 cells. Cells were treated at different concentrations of treatments for 3 h and stimulated with LPS for 24 h. The supernatants were collected and ELISA was performed according to the manufacturer’s protocol. Data were gathered from three separate experiments, each conducted in triplicate (n = 3). The data were analyzed by GraphPad Prism version 9.5.0 with one-way ANOVA and Dunnett’s multiple comparisons test (statistical significance denoted **** *p* < 0.0001 compared with cells treated with LPS).

Griess reagent was used to assess the effects of β-amyrin, stigmasterol, and the YTPS formulary extract on NO production in LPS-induced RAW 264.7 cells. Nitric oxide release was reduced slightly as the concentrations of β-amyrin and stigmasterol increased, as shown in Figure 4. The YTPS formulary extract markedly diminished the NO level in a dose-dependent manner. The concentrations of β-amyrin, stigmasterol, and the YTPS formulary extract that could significantly reduce the NO level to 50% were 55.52 ± 24.40 µM, >570 µM, 24.76 ± 1.48 µg/mL and above, as illustrated in Figure 5. YTPS formulary extract inhibited NO secretion with an IC_50_ value of 24.76 ± 1.48 µg/mL. 

#### 2.5.2. Effects of YTPS Formulary Extract, β-AMYRIN, Stigmasterol, and 13 Individual Extracts on IL-1β Secretion

The assessment of anti-inflammatory activity was further performed using the ELISA technique to measure the pro-inflammatory cytokine release brought on by LPS from THP-1 cells, using BAY 11-7085 (5 mM) as a positive control. The non-toxic doses of β-amyrin (20 µM), stigmasterol (20 µM), YTPS formulary extract (1000 µg/mL), *P. roxburghii* extract (100 µg/mL), *S. siamea* extract (100 µg/mL), *B. solanifolium* extract (100 µg/mL), *C. nardus* extract (100 µg/mL), *T. indica* extract (100 µg/mL), *M. azedarach* extract (100 µg/mL), *B. rotunda* extract (50 µg/mL), *A. ascalonicum* extract (100 µg/mL), *A. galanga* extract (100 µg/mL), *P. nigrum* extract (100 µg/mL), *A. sativum* extract (100 µg/mL), and a combination of *A. vera* and *F. assa-foetida* (100 µg/mL) demonstrated a notable range of impact on lowering IL-1β secretion from THP-1 cells. In comparison to cells treated with LPS, β-amyrin and stigmasterol, at a concentration of 20 µM, exhibited significant inhibitory effects on IL-1β secretion. YTPS formulary extract at 1000 µg/mL had no statistically significant effect on the secretion of IL-1β, whereas 500 µg/mL increased the secretion of IL-1β. The release of IL-1β, a cytokine that promotes inflammation, experienced a remarkable decrease when exposed to extracts of *P. roxburghii*, *S. siamea*, *C. nardus*, *M. azedarach*, *A. sativum*, *A. galanga*, *P. nigrum*, *A. ascalonicum*, and mixture of *A. vera* in combination with *F. assa-foetida* at a concentration of 100 µg/mL. This effect was observed, including 50 µg/mL of *T. indica* extract and *B. rotunda* extract, except for *B. solanifolium*, which stimulates IL-1β secretion. The IL-1β secretion of each intervention is illustrated in Table 3 and Figure 5. 

#### 2.5.3. Effects of the YTPS Formulary Extract, β-Amyrin, Stigmasterol, and 13 Individual Extracts on IL-6 Secretion

**Figure 6:**
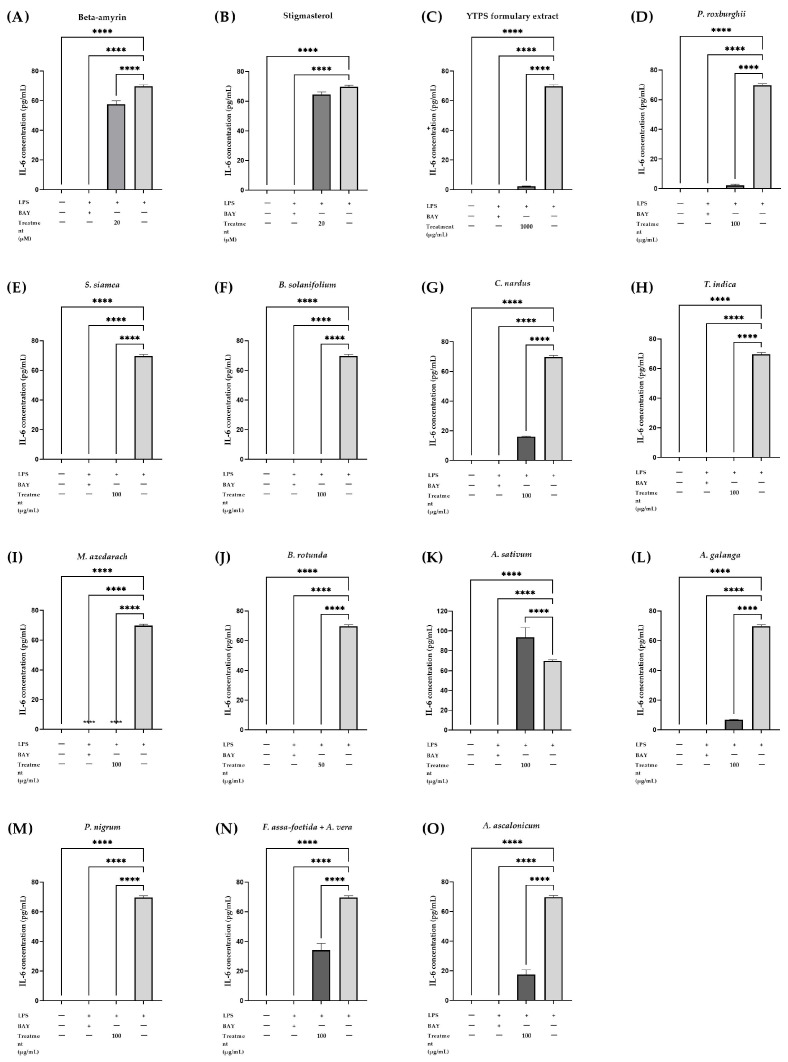
Effects of (**A**) β-amyrin (20 µM), (**B**) stigmasterol (20 µM), (**C**) YTPS formulary extract (1000 µg/mL), and (**D**–**O**) individual extracts (50 or 100 µg/mL) tested at non-toxic concentrations on the IL-6 release in THP-1 cells. Cells were treated at different concentrations of treatments for 3 h and stimulated with LPS for 24 h. The supernatants were collected and ELISA was performed according to the manufacturer’s protocol. Data were gathered from three separate experiments, each conducted in triplicate (n = 3). The data were analyzed by GraphPad Prism version 9.5.0 with one-way ANOVA and Dunnett’s multiple comparisons test (statistical significance denoted by **** *p* < 0.0001, compared with cells treated with LPS).

Most of the plant ingredients in the YTPS recipe, i.e., *P. roxburghii* extract (100 µg/mL), *S. siamea* extract (100 µg/mL), *B. solanifolium* extract (100 µg/mL), *C. nardus* extract (100 µg/mL), *T. indica* extract (100 µg/mL), *M. azedarach* extract (100 µg/mL), *B. rotunda* extract (50 µg/mL), *A. ascalonicum* extract (100 µg/mL), *A. galanga* extract (100 µg/mL), *P. nigrum* extract (100 µg/mL), *A. sativum* extract (100 µg/mL), and a combination of *A. vera* and *F. assa-foetida* (100 µg/mL), exhibited potent inhibition of IL-6 release from LPS-stimulated THP-1 cells. BAY 11-7085, at a concentration of 5 mM, served as the positive control for inhibiting IL-6 secretion in this experiment. In contrast to the cells treated with LPS, β-amyrin and stigmasterol, at a concentration of 20 µM, displayed significant inhibitory effects on IL-6 secretion. At concentrations of 500 and 1000 µg/mL, the YTPS formulary extract showed a substantial inhibitory effect in a dose-dependent manner on IL-6 secretion reduction. IL-6 release inhibition was observed when cells were treated with the extract of *P. roxburghii*, *S. siamea*, *B. solanifolium*, *C. nardus*, *M. azedarach*, *B. rotunda*, *A. ascalonicum*, *A. galanga*, *P. nigrum*, and mixture of *A. vera* in combination with *F. assa-foetida* at a concentration of 100 µg/mL. This impact was also observed in the treatment of 50 µg/mL of *T. indica* extract. Still, no reduction in IL-6 was noted when THP-1 cells were treated with *A. sativum* extract, which was evaluated at 100 µg/mL concentrations, as shown in Table 3 and Figure 6.

#### 2.5.4. Effects of YTPS Formulary Extract on TNF-α Secretion

**Figure 7:**
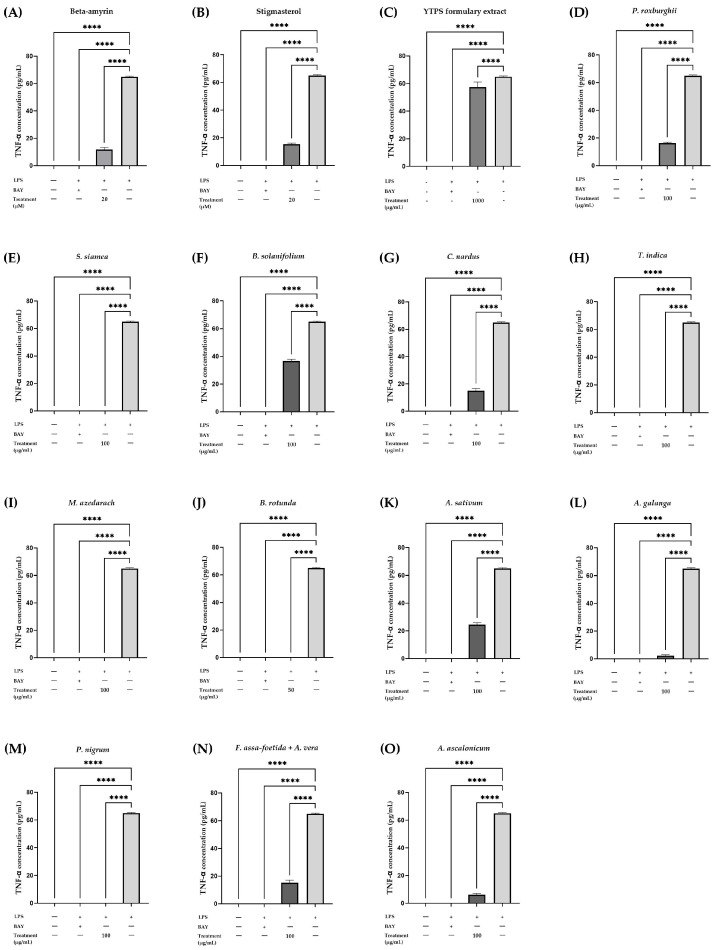
Effects of (**A**) β-amyrin (20 µM), (**B**) stigmasterol (20 µM), (**C**) YTPS formulary extract (1000 µg/mL), and (**D**–**O**) individual extracts (50 or 100 µg/mL) tested at non-toxic concentrations on the TNF-α release in THP-1 cells. Cells were treated at different concentrations of treatments for 3 h and stimulated with LPS for 24 h. The supernatants were collected and ELISA was performed according to the manufacturer’s protocol. Data were gathered from three separate experiments, each conducted in triplicate (n = 3). The data were analyzed by GraphPad Prism version 9.5.0 with one-way ANOVA and Dunnett’s multiple comparisons test (statistical significance denoted by **** *p* < 0.0001 compared with cells treated with LPS).

The analyzed compounds displayed a considerable range of influence on the secretion of TNF-α. β-amyrin and stigmasterol, at a concentration of 20 µM, displayed significant inhibition of TNF-α secretion compared to THP-1 cells stimulated with LPS. At concentrations of 500 and 1000 µg/mL, YTPS formulary extract notably boosted TNF-α secretion by at least 30%. Inhibition of TNF-α release was observed upon exposure to the extracts of *P. roxburghii*, *S. siamea*, *C. nardus*, *M. azedarach*, *A. ascalonicum*, *A. galanga*, and *P. nigrum*, as well as a mixture of *A. vera* in combination with *F. assa-foetida* at a concentration of 100 µg/mL. The same effect was observed in THP-1 cells treated with 50 µg/mL of *B. rotunda* extract. No alterations in TNF-α secretion were observed with 100 µg/mL of *A. sativum* extract. The extract of *B. solanifolium* induced the secretion of TNF-α at a concentration of 50 µg/mL, nearly 50%. Table 3 and Figure 7 illustrate the TNF-α release for each treatment. 

### 2.6. Safety Evaluation of the YTPS Formulary Extract

#### 2.6.1. Safety Evaluation on the Skin Using HaCaT Cells 

**Figure 8:**
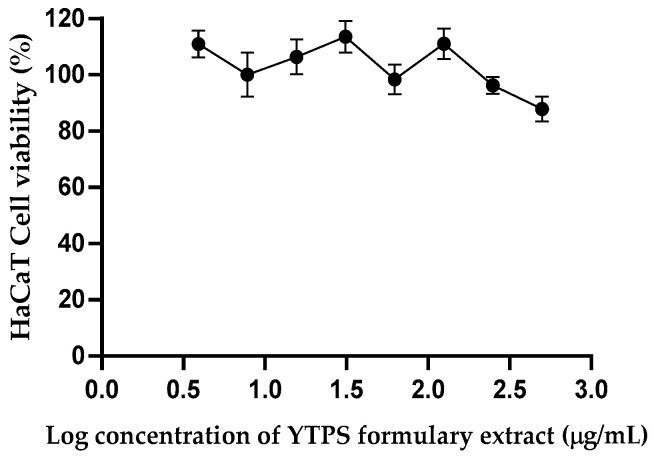
Effect of the YTPS formulary extract (3.91–500 µg/mL) on the cell viability of HaCaT cells. Cells were treated at different concentrations for 24 h and treated with MTT reagent for 2 h, and data are expressed as a percentage of control (media only). Data were gathered from three separate experiments; each conducted with triplicate wells (n = 3). The descriptive statistics were analyzed by GraphPad Prism version 9.5.0.

Since the YTPS formulary was used as a local pain reliever by applying it to the skin, the effect of the YTPS formulary extract on the skin was tested on the human keratinocyte cell line, HaCaT cells. After a 24 h incubation period, the impact of the different YTPS formulary extract concentrations on the viability of HaCaT cells was assessed. The maximum concentration of herbal extract tested was within the limit that Song et al. tested [17]. As shown in Figure 8, following the administration of the YTPS formulary extract at concentrations ranging from 3.91 to 500 µg/mL, no appreciable cytotoxicity was seen. The IC_50_ of cell viability for HaCaT cells was >500 µg/mL.

#### 2.6.2. Safety Evaluation on the Kidney Using HEK293 Cells

**Figure 9:**
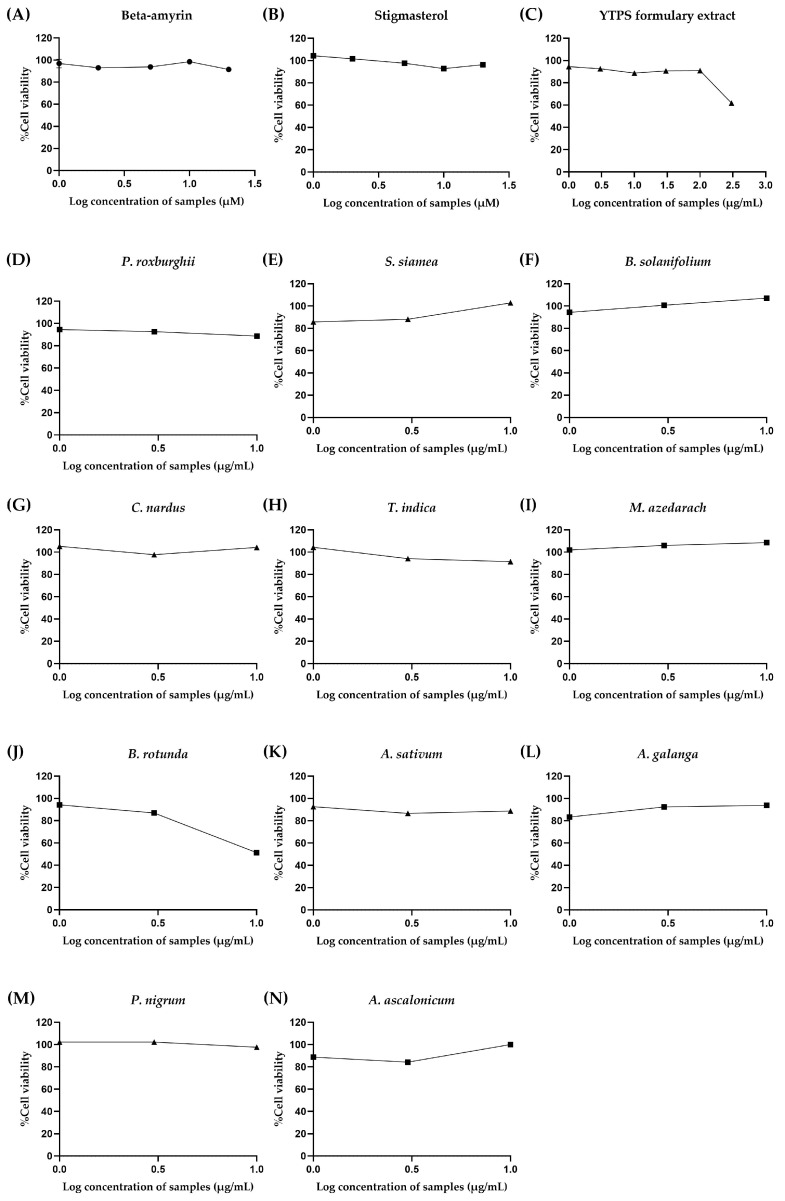
Effects of (**A**) β-amyrin (1–20 µM), (**B**) stigmasterol (1–20 µM), (**C**) YTPS formulary extract (1–300 µg/mL), and (**D**–**N**) individual extracts (10–100 µg/mL) on the cell viability of HEK293 cells. Cell viability was assessed using an Alamar blue metabolic assay by treating the cells with Resazurin dye solution for 4 h after 24 h of incubation of samples. Alamar blue fluorescence was measured, and data are expressed as a percentage of control (media only). Supernatants were collected and measured with FlexStation^®^ 3 at 570 nm excitation and 600 nm emission using SoftMax Pro 5.4.6 software for data recording and analysis. Data were gathered from three separate experiments, each conducted in triplicate (n = 3). The descriptive statistics were analyzed by GraphPad Prism version 9.5.0.

Nephrotoxicity was one of the most common side effects found in patients using NSAIDs. HEK293 cells were used as a surrogate for a kidney cell toxicity test. We tested a similar maximum extract concentration as Grauzdytė et al. [18]. β-amyrin, stigmasterol, YPTS extract, and individual extract concentrations were varied for an AlamarBlue assay: 1–20 µM, 1–20 µM, 1–300 µg/mL, and 10–100 µg/mL, respectively. The cells were incubated with the samples for 24 h prior performing the assay. There was no significant toxicity found in the cells, except for *B. rotunda* extract at the concentration of 100 µg/mL, which decreased cell viability to nearly 50%, as shown in Figure 9. The IC_50_ values of samples after treatment with HEK293 cells can be found in Supplementary Table S1.

## 3. Discussion

In this study, the bioactive compounds in the YTPS formulary extract and individual herbal composition in the YTPS formulary extract were employed to investigate cytotoxicity and anti-inflammatory activities. The evaluation focused on specific pro-inflammatory cytokine secretion, namely, IL-1β, IL-6, and TNF-α. These cytokines are essential for managing osteoarthritis [19]. Additionally, the investigation also measured the NO level, given its involvement in conveying the consequences of several pro-inflammatory cytokines, including IL-1β and TNF-α, blocking the production of collagen and proteoglycans and upregulating the matrix metalloproteinases (MMPs) enzyme [20]. Cytotoxicity was evaluated in human embryonic kidney and human leukemia monocytic cell lines. The THP-1 cell line was applied for the anti-inflammatory evaluation model for its role in pro-inflammatory cytokine production [21]. The Key results obtained from the identification and quantification of bioactive compounds and biological activities of the YTPS extract can be found in Supplementary Figure S1.

From the previous study, screening of YTPS formulary extract was conducted to identify markers for the purpose of quantifying the compounds responsible for their anti-inflammatory effects. It was found that β-amyrin and stigmasterol were the major compounds found in the YTPS formulary extract, according to the main component of the formulary, *P. roxburghii* [22]. β-amyrin [23], a pentacyclic triterpenoid [24], was found in several plants, for example, *Alstonia boonei* De Wild. [9], *Protium heptaphyllum* (Aubl.) Marchand [25], *Myrcianthes pungens* (O. Berg) D. Legrand [26], and *Hibiscus calyphyllus* Cav. [27]. The compound possessed anti-inflammatory activity [28]. The anti-inflammation of β-amyrin was contributed by COX, LOX, and NOS inhibition, including the reduction in PGE_2_ and IL-6 secretion [12]. Among the frequently found plant sterols, stigmasterol [29], a tetracyclic triterpene, also exhibits anti-osteoarthritis and anti-inflammatory properties [10]. It was found that stigmasterol inhibits IL-1β, IL-6, TNF-α, NO, and COX-2 production [30]. Stigmasterol is found in several plants, for instance, *Hygrophila schulli* M.R. Almeida & S.M. Almeida [31], *Wrightia tinctoria* R. Br. [32], and *Neocarya macrophylla* (Sabine) Prance ex F. White [33]. 

There is evidence linking oxidative stress and inflammation. Oxidative stress, caused by overproduction of reactive oxygen species (ROS), leads to significant cellular and tissue damage and promotes chronic inflammation [34,35]. In addition, oxidative stress can activate NF-κB and activator protein-1 (AP-1) and contribute to gene transcription of growth factors, extracellular matrix (ECM) proteins, and cytokines, such as IL-1β, IL-6, IL-18, and TNF-α [36]. A recent study found that dietary components with high antioxidant capacities, specifically FRAP and DPPH levels, can lower inflammatory and oxidative stress biomarkers [37]. Notably, the antioxidant properties of β-amyrin have been evidenced in several studies. β-amyrin can scavenge free radicals and increase antioxidant enzymes, namely, glutathione peroxidase (GPx), reduced glutathione (GSH), superoxide dismutase (SOD), and catalase (CAT) [38]. Stigmasterol has been extensively investigated for antioxidant capacity *in vitro* and *in vivo*. It diminishes the production of ROS and mitigates oxidative damage by bolstering both enzymatic and non-enzymatic antioxidant systems [10]. Furthermore, stigmasterol synergistically collaborates with phytosterols in reducing ROS. This reduction also extends to certain oxidoreductase enzymes, which are significant contributors to cellular oxidative stress [39]. 

The inflammation in osteoarthritis is regulated by biochemical substances, such as prostanoids, cytokines (IL-1α, IL-1β, TNF-α, PGE_2_), proteases, and ROS produced by synoviocytes and chondrocytes. ROS are crucial to the deterioration of tissues, including inflammatory response, as it reacts with proteins, lipids, and DNA due to its oxidizing ability. Long-term effects of ROS production contribute to the progression of inflammatory disease [40]. Apart from the inflammation effect, ROS act as secondary messengers in pathological conditions and causes degradation from oxidation reactions [41]. Moreover, the ROS level is increased and antioxidant enzymes are diminished in osteoarthritis patients [40], suggesting that ROS has a vital role in inflammation in osteoarthritis.

Numerous studies support the anti-inflammatory and antioxidant qualities of phenolic compounds and flavonoids. Flavonoids stabilize the structure of reactive free radicals, enabling the effects to be less reactive, thereby making them efficient scavengers of such radicals [42]. These polyphenols also modulate immune cells, cytokine production, and pro-inflammatory gene expression. The ability to alleviate inflammatory cytokines such as IL-1β, IL-6, IL-8, IL-17, TNF-α, and IFN-γ of flavonoids has been demonstrated by many studies [43]. The YTPS ethanolic extract showed antioxidant activities, with 144.50 ± 2.82 µg/mL and 31.85 ± 0.18 µg/mL, of 50% inhibition by DPPH and ABTS, respectively. The FRAP value was 655.4 ± 19.11 µM at 1250 µg/mL. The antioxidant effects of the YTPS formulary extract were correlated to both phenolic and flavonoid contents. Phenolic compounds are commonly found in herbal extracts. Several studies have discovered anti-inflammatory properties as the phenolic compounds act similarly to NSAIDs, in addition to inhibition of PGE_2_ production; nitric oxide synthase; COX-2; and pro-inflammatory mediators and/or gene expressions such as IL-6, IL-8, TNF-α, and IL-1β [44]. Likewise, flavonoid compounds act on many distinct mechanisms, for instance, NF-κB transcription factor, COX, LOX, and Phospholipase A_2_ (PLA_2_) inhibitor, resulting in decreases in pro-inflammatory cytokine, prostaglandin, thromboxane, and leukotriene production. Flavonoids additionally inhibit protein kinases associated with the signaling cascade that triggers the activation of inflammatory processes [45]. The YTPS ethanolic extract was analyzed for both total phenolic content and total flavonoid content for the first time. The values were found to be 117.73 ± 8.55 mg GAE/g extract and 3148.06 ± 2.05 mg QAE/g extract for total phenolic content and total flavonoid content, respectively. These results indicate that the β-amyrin, stigmasterol, and phenolic and flavonoid compounds contained in the mixture of YTPS formulary extract, a polyherbal formulary, contribute to the anti-inflammatory activity.

For an investigation of the safety and biological activity of YTPS extract, cell viability was evaluated in several cell lines—HaCaT, RAW264.7, HEK293, and THP-1 cells—to find a non-toxic concentration that cells could be exposed to. Varied concentrations of treatments were added to each cell line, including β-amyrin, stigmasterol, the YTPS formulary extract, and individual herbal extract, to determine the cytotoxicity. The human keratinocyte cell line was employed for skin cytotoxicity evaluation since YTPS is a topical spray formulation. The human embryonic kidney cell line was used as a representative for the evaluation of adverse effects on renal function. The macrophage and monocyte were used as surrogates for testing anti-inflammatory properties due to their ability to secrete inflammatory cytokines [46]. The findings showed that when the concentration of YTPS floral extract rose, cell viability decreased. However, the highest concentrations of YTPS ethanolic formulary extract at 500, 250, 300, and 1500 µg/mL significantly impaired cell viability on the HaCaT, RAW264.7, HEK293, and THP-1 cell lines, respectively. Nevertheless, the YTPS formulary extract demonstrated an elevation in the cell viability of THP-1 when the concentration ranged between 137 and 750 µg/mL before returning to the same as the control level at 1500 µg/mL. A similar result was observed with *P. roxburghii*, the primary herbal ingredient in the YTPS formulation, at a 100 µg/mL concentration. Our findings indicated that the increase in cell viability in the YTPS formulary treatment could be attributed to *P. roxburghii* extract. Other individual extracts at the highest concentration of 100 µg/mL were considered non-toxic to all tested cell lines except *B. rotunda* extract, which led to a decrease in cell viability to less than 80% in the HEK293 and THP-1 cell lines. 

Macrophages and monocytes play essential roles in the inflammatory process, which is one of the factors affecting the progression of knee osteoarthritis. Both cells are suitable surrogates for testing anti-inflammatory properties due to their ability to produce and secrete inflammatory cytokines. THP-1 cells are widely used as an *in vitro* model for human monocytes and macrophages in mechanistic studies of inflammatory diseases. In *in vitro* experiments, THP-1 cells can be activated by stimulating them with inflammatory activators, such as LPS or pro-inflammatory cytokines. Chanput et al. and Sharif et al. have shown that THP-1 cells exposed to LPS exhibit changes in the expression of several inflammation-related genes, including IL-1β, IL-6, IL-8, IL-10, and TNF-α [47,48]. RAW264.7, a monocyte/macrophage-like cell line, is also the most commonly used *in in vitro* studies for screening anti-inflammatory activity from natural compounds. LPS, a common inducer derived from *Escherichia coli*, can upregulate numerous inflammatory mediators such as NO, COX-2, TNF-α, and IL-6 in RAW264.7 cells during the inflammatory response [49]. RAW264.7 is a macrophage cell that has a major role in inflammation diseases [50]. Recent studies have shown that macrophages are also the key to structural progression and symptomology, including initiation and progression of osteoarthritis. The macrophage is regulated by NF-κB signaling pathway, which is found in both cell lines [51]. In addition, both cell lines can produce the three main pro-inflammatory cytokines that are significantly elevated in osteoarthritis patients, namely, IL-1β, IL-6, and TNF-α [52]. Apart from the rational use of the cell lines, they require low maintenance, and the growth rate is considerable high [50]. Moreover, there was a study on the anti-inflammatory effects of herbal fractions on both cell lines [53].

Nitric oxide plays an important role as a signaling molecule, triggering many effects on human physiological functions. These encompass the regulation of blood pressure, blood circulation, platelet activity, inflammatory responses, neurotransmission, and immune functions. Moreover, NO has significant negative effects on osseous tissue, including osteoclasts, osteoblasts, and chondrocytes, resulting in the progression of osteoarthritis. NO can bind with superoxide anions, which causes cartilage structures to become inflamed and leads to cell death. Additionally, NO induces a persistent release of inflammatory factors, exacerbating the disease process. Furthermore, inflammatory mediators and the ROS/RNOS system serve as primary initiators of the inflammatory response. Their interaction with NO creates a positive feedback loop, perpetuating the release of inflammation-associated molecules and exacerbating damage to cartilage tissues [54]. NO also regulates inflammation responses by a variety of signaling pathways, for instance, Janus kinase/signal transducers and activators of transcription (Jak-STAT), AP-1, and NF-κB. Activation of NF-κB by NO plays a critical role in the inflammation response [55], including the increase in inflammatory cytokine production, IL-1, IL-2, IL-6, IL-8, IL-12, and TNF-α [56]. NO, a highly reactive free radical, is a second messenger that triggers an inflammation response. An *in vivo* study showed that macrophages are activated *via* the NF-κB signaling pathway and produce NO during inflammation [57]. RAW264.6 cells are macrophages which are frequently used as a model for anti-inflammatory studies, and are tested using the NO assay [58,59,60]. We found that the IC_50_ value of the YTPS formulary extract obtained from NO secretion inhibition was 24.76 ± 1.48 µg/mL; it dramatically decreased NO synthesis in RAW264.7 cells in a dose-dependent manner. Therefore, these data demonstrate that the YTPS formulary extract shows promise as a potential alternative for alleviating inflammation. 

Many factors contribute to the progression of osteoarthritis; one of the vital considerations is pro-inflammatory cytokines. In addition to the enhancement of degeneration, these cytokines also disturb the homeostasis of joint tissue metabolism. Three main pro-inflammatory cytokines that engage in the pathophysiology of osteoarthritis are IL-1β, IL-6, and TNF-α [52]. IL-1β triggers inflammatory responses and catabolic effects both independently and in collaboration with other mediators, impacting joint cartilage and other components of joints. The progression involves the NF-κB signaling pathway, triggered by IL-1β, affecting the enzymes linked with pathophysiology of osteoarthritis, including iNOS, PLA_2_, COX-2, prostaglandin E synthase 2 (PGE_2_ synthase), and MMPs [19]. This pathway also promotes the synthesis and release of pro-inflammatory cytokines like IL-6 and TNF-α. Additionally, NF-κB activation by IL-1β induces the synthesis of chemokines such as IL-8, MCP-1, CCL5, and MIP-1a, which attract inflammatory cells, exacerbating joint inflammation [61]. Joint disorders that are inflammatory and degenerative are associated with IL-6 and sIL-6R. The synovial fluid and sera of patients suffering from osteoarthritis and rheumatoid arthritis also have higher concentrations of IL-6 and sIL-6R. IL-6, in conjunction with IL-1β and oncostatin, increases MMP-1 and MMP-13 expression while lowering type II collagen expression [52]. Moreover, its impact involves promoting the formation of osteoclasts, leading to bone resorption, particularly in collaboration with IL-1β and TNF-α. Osteoblasts, when activated by IL-1β, TNF-α, and IL-6, not only contribute to this process but may also generate MMPs, potentially harming nearby cartilage. TNF-α, in combination with IL-1β, is recognized as a pivotal inflammatory cytokine implicated in the pathophysiological mechanisms observed during the progression of osteoarthritis [19]. Activation of similar signaling pathways as IL-1β leads to a synergistic effect between these two cytokines. TNF-α inhibits chondrocyte production in proteoglycan components and type II collagen, contributing to ECM degradation by inducing collagenases and aggrecanases like MMP-1, MMP-3, MMP-13, and ADAMTS-4 in collaboration with IL-1β. This process elevates the breakdown of joint tissues due to complex 2 signaling pathway activation and subsequent cell apoptosis. Additionally, TNF-α enhances the synthesis of IL-6. Alongside IL-1β, TNF-α promotes the generation of iNOS, COX-2, and PGE_2_ synthase, further amplifying IL-1β and TNF-α production [61]. LPS, which is present in the outer membrane of gram-negative bacteria, was used in this experiment to trigger the release of pro-inflammatory mediators from monocytes and macrophages such as IL-1β, IL-6, and TNF-α by binding to Toll-like receptors 4 (TLR4) [62]. While LPS has the ability to trigger the cytokine production that promotes inflammation *via* monocytes and macrophages, the development of joint inflammation in osteoarthritis results from a multitude of intricate pathological signaling pathways and crucial molecules implicated in the pathogenesis of osteoarthritis [63]. Therefore, it is important not to discount the potential involvement of other signaling pathways. 

Activation of an NF-κB signaling pathway in monocytes and macrophages leads to the production of several pro-inflammatory cytokines, for example, IL-1β, IL-2, IL-6, IL-8, IL-12, and TNF-α [64]. BAY 11-7085, an inhibitor of NF-κB activation [65], was used as a positive control for the anti-inflammatory assay. THP-1 cells underwent treatment with β-amyrin, stigmasterol, and the YTPS formulary extract, and individual herbal extracts were evaluated with pivotal cytokines contributing to the progression of osteoarthritis, IL-1β, IL-6, and TNF-α. The YTPS formulary extract at 1000 µg/mL slightly diminished IL-1β release. Both major compounds of YTPS formulary extract, β-amyrin and stigmasterol, along with individual herbal extracts, namely, *P. roxburghii*, *S. siamea*, *B. solanifolium*, *C. nardus*, *T. indica*, *M. azedarach*, *A. sativum*, *A. galanga*, *P. nigrum*, *F. assa-foetida*, *A. vera*, *A. ascalonicum*, significantly reduced IL-1β secretion from LPS-stimulated THP-1 cells. With the decline in IL-1β, COX-2 activity decreased via the NF-κB signaling pathway [66]. β-amyrin significantly decreased IL-1β, IL-6, and TNF-α secretion. This is the first time it has been shown that β-amyrin can decrease IL-1β production. The decreases in IL-6 and TNF-α from β-amyrin were previously reported by Melo et al. [67]. The effect of stigmasterol on the IL-1β secretion agreed with Mo et al. [68], who found that the compound down-regulated the sterol regulatory element-binding transcription factor 2 (SREBF2) and promoted the inhibitory effect of ferroptosis inhibitors. Stigmasterol also inhibited TNF-α, which correlates with the studies reported by Kangsamaksin et al. and Gabay et al. showing that it downregulates TNF-α expression [69,70]. 

In addition to the anti-inflammatory benefits of the mixed herb YTPS formulary extract, several of the individual components provide the same consequences. *C. nardus.* significantly reduces IL-1β production, possibly attributable to the presence of citronellol. Grazul et al. discovered that citronellol can diminish IL-1β production *in vivo* [71]. According to Miao et al., luteolin, a compound found in *T. indica*, decreases IL-1β, IL-6, and TNF-α by inhibiting the PPAR-γ/STAT/MyD88 pathway [72]. The reduction in IL-1β and IL-6, found in *S. siamea*, *M. azedarach*, *A. ascalonicum*, *A. galanga*, *P. nigrum*, *F. assa-foetida*, *A. vera,* and *B. rotunda* extract, suppresses activation of the receptor activator of NF-κB ligand (RANKL), which reduces the differentiation of osteoclast and bone resorption. Moreover, the inhibition of the NF-κB signaling pathway by IL-1β and TNF-α decreases the inflammation of joints and tissue degradation and prevents the effects of iNOS and MMP, which cause inflammation and articular cartilage breakdown in osteoarthritis [73]. These individual plants also block TNF-α release. The TNF-α plays a crucial role in inflammation through activation of MAPK and the NF-κB signaling pathway, leading to the upregulation of pro-inflammatory genes. Thus, the blockade of TNF-α reduces the inflammatory effects [74]. To the best of our knowledge, there has been no report of the effects on the pro-inflammatory cytokine of *S. siamea*, *B. solanifolium*, and *M. azedarach* before. The results of *B. rotunda,* which lowered IL-1β and TNF-α secretion, were comparable to the experiment performed by Voon et al. [75]. The same inhibitory effects of *A. ascalonicum* on all pro-inflammatory cytokines were studied by Werawattanachai et al. [76]. In accordance with George et al., *A. galanga* inhibited the pro-inflammatory cytokine by modulation of the TLR4 and JAK/STAT pathway [77]. Duan et al. revealed that piperine, a prominent compound detected in *P. nigrum*, modulates the NF-κB and MAPK signaling pathways for its inhibitory pro-inflammatory cytokine production [78]. Allicin, a substance from *A. sativum*, was discovered by Liu S et al. as an IL-6 and TNF-α inhibitor by blocking the activation of a p38 signaling pathway [79]. In addition, Samra et al. discovered that allicin suppresses IL-1β release by inhibiting NLRP3 inflammasome activation [80]. Nonetheless, our data found that *A. sativum* and *B. solanifolium* had no inhibitory effects on IL-6 and IL-1β production, respectively. Xing et al. revealed that *F. assa-foetida*, one of the plants that belongs to the *Ferula* species, could reduce the expression of IL-1β, IL-6, and TNF-α [81]. *A. vera* has the ability to decrease IL-1β, IL-6, and TNF-α production, as discovered by Yun et al. [82]. The decrease in IL-1β production in the YTPS formulary extract could be due to a synergistic effect of β-amyrin, stigmasterol, and individual herbal extract. 

The YTPS formulary extract is a polyherbal mixture composed of 13 herbal ingredients. The major component of the formulary, comprising 37% of the recipe, is *P. roxburghii.* The literature reviews show that β-amyrin and stigmasterol are two major markers possessing anti-inflammatory activity [9,10]. In our study, the YTPS formulary ethanolic extract contained 0.25 mg/g of β-amyrin and 0.03 mg/g of stigmasterol. The anti-inflammatory property was contributed by the NO levels and two pro-inflammatory cytokines, IL-6 and TNF-α. Due to inflammation, NO production increased through the NF-κB signaling pathway [57], which regulates three pro-inflammatory cytokines of osteoarthritis, including IL-1β, IL-6 and TNF-α [66]. The YTPS formulation suppressed NO levels; hence, a second messenger that triggers the inflammation response was diminished. The reduction in the NO, IL-6, and TNF-α effects in YTPS ethanolic formulary extract agreed with Mangmool et al. regarding the inhibition of iNOS, COX-2, and TNF-α mRNA expression after YTPS ethanolic formulary extract was employed in the RAW264.7 cells [83]. The YTPS formulary extracts substantially lowered IL-6 cytokine release in a dose-dependent manner; this effect was observed along with β-amyrin and individual extracts comprising *P. roxburghii*, *S. siamea*, *B. solanifolium*, *C. nardus*, *T. indica*, *M. azedarach*, *B. rotunda*, *A. ascalonicum*, *A. galanga*, *P. nigrum*, *A. vera*, and *F. assa-foetida*. The TNF-α release was blocked by the YTPS formulary extract, β-amyrin, stigmasterol, *P. roxburghii*, *S. siamea*, *B. solanifolium*, *C. nardus*, *T. indica*, *M. azedarach*, *B. rotunda*, *A. ascalonicum*, *A. galanga*, *P. nigrum*, *A. sativum*, *A. vera*, and *F. assa-foetida*. On the other hand, the YTPS formulary extract had no effects on IL-1β secretion. However, β-amyrin, stigmasterol, *P. roxburghii*, *S. siamea*, *C. nardus*, *T. indica*, *M. azedarach*, *A. ascalonicum*, *A. galanga*, *P. nigrum*, *A. sativum*, *A. vera*, and *F. assa-foetida* were found to reduce IL-1β release notably. From our study, beta-amyrin and stigmasterol significantly inhibited LPS-induced IL-1β and TNF-α. In contrast, treatment with YTPS formulary extract had few inhibitory effects owing to the small amount found in the YTPS formulary extract: 70 nM and 606 nM, respectively. Although this study reveals the first data regarding all the individual herbal components in the YTPS formulary and their anti-inflammatory effects, our limitations include a lack of exploration of additional bioactive compounds, different inflammation signaling pathways, or mediators, as well as a confirmation of gene expression of each anti-inflammatory assay. Intriguingly, the YTPS formulary extracts completely inhibited LPS-induced IL-6. In contrast, the treatment with beta-amyrin and stigmasterol had no effects. Since the YTPS formulary extract presents a polyherbal mixture, the anti-inflammatory effects may be the consequence of other substances in ingredients of the formulary that can inhibit IL-6 production, for example, piperine, pinostrobin, aloesin, and aloe-emoldin. These results suggest that many compounds act synergistically to reduce inflammatory cytokines through varied signaling pathways, so the YTPS formulary should be used in the polyherbal combination to maximize its efficacy. 

The risks of conventional treatment for pain and inflammation in osteoarthritis patients include adverse GI and renal function effects, especially with long-term use. From this experiment, our findings suggest that the YTPS ethanolic extract exerts anti-inflammatory properties through the reduction in IL-6 and TNF-α secretion, the two main pro-inflammatory cytokines found at high levels while the inflammation of knee osteoarthritis occurs. Moreover, the formulary had no cytotoxicity against kidney cells, and could be used in long-term treatment for chronic inflammatory disease. Therefore, the YTPS ethanolic extract could be used as an alternative remedy or an adjunctive to the standard treatment or topical formulation owing to the YTPS extract’s good anti-inflammatory effect with low toxicity.

## 4. Materials and Methods

### 4.1. Materials

β-Amyrin and stigmasterol reference standards were purchased from ChemFaces (Wuhan, China). Absolute ethanol (analytical grade), acetonitrile (HPLC grade), methanol (HPLC grade), water (HPLC grade), glacial acetic acid, sodium nitrite, aluminum chloride, ferric chloride, ferrous sulfate, potassium persulfate, L-ascorbic acid, gallic acid, and quercetin were bought from Daejung Chemical Co. (Busan, Republic of Korea). DPPH, ABTS, and sodium acetate were sourced from Sisco Research Laboratories (Mumbai, India). Folin and Ciocalteu’s phenol reagent, bovine serum albumin, hydrochloric acid, resazurin sodium salt, dimethyl sulfoxide, and gallic acid were purchased from Sigma-Aldrich (St. Louis, MO, USA). Trolox^®^ was acquired from Thermo Scientific Chemicals (Waltham, MA, USA). RPMI medium 1640, DMEM/F-12 (1:1), 0.25% trypsin-EDTA, and penicillin–streptomycin were purchased from Gibco (Waltham, MA, USA). Tween^®^ 20, sodium hydroxide, phosphate-buffered saline tablets, and sodium chloride were purchased from Fisher (Waltham, MA, USA). Fetal bovine serum was bought from PAN Biotech (Aidenbach, Germany). IL-1β and TNF-α ELISA kits were purchased from BD Biosciences (San Jose, CA, USA). An IL-6 ELISA kit was bought from R&D Systems (Minneapolis, MN, USA). HaCaT (EP-CL-0090; Elabscience, Houston, TX, USA), and RAW264.7 cells were obtained from ATCC (Manassas, VA, USA). HEK293 (PCS-200-011) and THP-1 (TIB-71) cells were acquired from Associate Professor Leanne Strokes and Professor Maria O’Connell, School of Pharmacy, the University of East Anglia, the United Kingdom.

### 4.2. Methods

#### 4.2.1. Plant Material and Extraction Procedure

All plants listed in Table 4 were collected from their localities in Thailand and identified by Mr. Nopparut Toolmal, a plant taxonomist. Only *F. assa-foetida*, *A. vera*, and *A. ferox* was purchased from a Thai traditional drug store. The voucher specimens were deposited at the Thai Traditional Medicine Herbarium (TTM), Bangkok, Thailand. The part of each herbal composition used in the YTPS formulary was described in the National Thai Traditional Medicine Formulary book of the ministry of public health, Thailand. This information was appraised by an expert committee comprising an ancient linguist, a Thai traditional medicine doctor, a botanist, a pharmacist, and a medical doctor.

#### 4.2.2. Preparation of Plant Extracts

**Figure 10:**
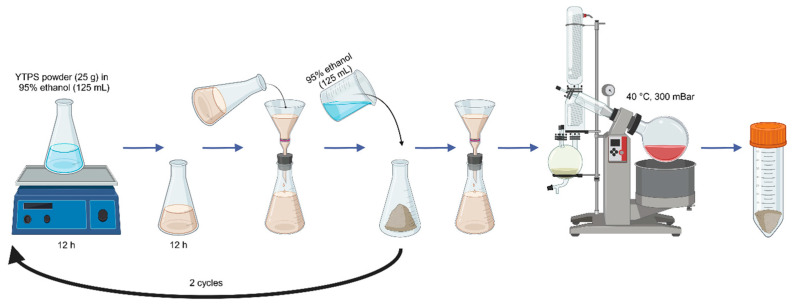
The extraction process of YTPS formulary and individual plant ingredients. The dried powder (300 g) was macerated with 95% ethanol (3000 mL), filtered, and evaporated until dry.

To ensure that the right parts were used, all foreign matters were dismissed from the collected specimens. Each YTPS herbal component was cleaned, cut, and dried at 40 °C in a hot air oven (Binder^®^, BINDER GmbH, Tuttlingen, Germany) overnight until completely desiccated. The dried plant materials in the YTPS formulation were milled, sieved, and proportionally weighed as described in Table 1, then dry mixed. The YTPS mixed powder (300 g) and individual plant ingredients were macerated with 95% ethanol (3000 mL) and periodically shaken (orbital shaker, Hysc lab^®^, Hanyang Science Lab Co., Ltd., Seoul, Republic of Korea) for 48 h. After filtering the macerated samples through filter paper, they were evaporated in a vacuum oven at 40 °C with 300 millibar pressure until the macerate became dry, then refrigerated at 2 °C until use or analysis. The extraction process is illustrated in Figure 10.

#### 4.2.3. Determination of Total Phenolic Content of YTPS Formulary Extract

The Folin–Ciocalteu reaction was used to determine the total phenolic content of the YTPS formulary extract, following the protocol outlined by Chittasupho et al. [84]. The 96-well plates were prepared with gallic acid solution (ranging from 3.9 to 2000 µg/mL). YTPS formulary extract and each plant extract were tested at concentrations ranging from 3.9 to 5000 µg/mL. To each well, 100 µL of 10% *v*/*v* Folin–Ciocalteu phenol reagent was added, followed by 50 µL of 7.5% *w*/*v* sodium carbonate solution. After that, the plates were incubated for 2 h at room temperature with a light shield. The absorbance of the solutions was then determined using a microplate reader (FlexStation^®^ 3, Molecular Devices, LLC, San Jose, CA, USA) at a wavelength of 750 nm. Using a calibration curve of gallic acid, the total phenolic contents were calculated, and the results were represented as mg/g gallic acid equivalents of dried crude extract.

#### 4.2.4. Determination of Total Flavonoid Content of YTPS Formulary Extract

Following the protocol outlined by Chittasupho et al., the total flavonoid content of the YTPS formulary extract was evaluated using the aluminum chloride colorimetric method [84,85]. In this test, 30 µL of 5% *w*/*v* sodium nitrate was added to a 96-well plate after 100 µL of either quercetin solution (varying from 3.9 to 1000 µg/mL) or YTPS formulary extract and each plant extract (ranging from 3.9 to 5000 µg/mL) was added. After a 5-min incubation period, 50 µL of a 2% *w*/*v* aluminum chloride solution was applied and allowed to incubate for 6 min, followed by additional incubation for 10 min with 1 N sodium hydroxide. Subsequently, the absorbance was read at 510 nm. The acquired data were analyzed and calculated as mg/g of quercetin equivalents of dried crude extract.

#### 4.2.5. HPLC Analysis for the Identification and Quantification of Bioactive Compounds in YTPS Formulary Extract

β-amyrin and stigmasterol were two markers used for the identification and quantification of the YTPS formulary extract by the standard addition method using high-performance liquid chromatography (HPLC). The YTPS formulary extract was diluted with 95% ethanol, obtaining 10,000 µg/mL. The YTPS formulary extract solution was spiked with a mixed standard working solution, achieving 100 µg/mL of β-amyrin and stigmasterol. Before injection, the spiked sample underwent filtration with an injection volume of 20 µL. HPLC analysis was performed with the Thermo Scientific™ Vanquish™ LC Systems (Waltham, MA, USA) equipped with a Quaternary pump and a Hypersil GOLD™ C_18_ column (250 mm × 4.6 mm i.d.) with a 5 μm particle size, operated at room temperature. The mobile phase consisted of acetonitrile (A) and methanol (B) at a 96:4 ratio, with a flow rate of 1.5 mL/min. The photodiode array (PDA) was set at 202 nm for the detection wavelength. The contents of β-amyrin and stigmasterol were calculated using the calibration curve of the spiked sample with Chromeleon^TM^ (Thermo Scientific). β-amyrin and stigmasterol were determined based on their retention times corresponding to the reference standards in the chromatogram of the YTPS spiked extract. 

#### 4.2.6. Antioxidant Assay

##### 2,2-Diphenyl-1-picrylhydrazyl (DPPH) Radical Scavenging Activity Assay

The DPPH radical scavenging activity assay followed the protocol outlined by Athikomkulchai et al. [86]. The DPPH radical was dissolved in methanol. The positive controls were ascorbic acid, gallic acid, quercetin, Trolox^®^ (3.91–2000 µg/mL), and YTPS formulary and individual extract (9.77–5000 µg/mL). β-amyrin and stigmasterol, in the range of 1.95–1000 µg/mL, were dissolved in deionized water and 95% ethanol, respectively. A mixture of 100 µL of DPPH and 100 µL of different YTPS formulary extract concentrations was added to each well of a 96-well plate. The plate was then incubated at room temperature for 30 min. Following an incubation, the absorbance was measured with a microplate reader at 570 nm. The IC_50_ values were expressed as the concentration of the extract achieving a 50% reduction in the absorbance of DPPH compared to the negative control.

$$DPPH\;radical\;scavenging\;activity\;(\%)=\frac{A-B}{A}\times 100\%$$

In this equation, A represents the absorbance observed in the DPPH reaction with the solvent control, while B represents the absorbance recorded in the DPPH reaction with the extract.

##### 2,2′-Azino-bis (3-Ethylbenzthiazoline-6-sulphonic Acid (ABTS) Radical Scavenging Activity Assay

The ABTS radical scavenging activity assay was conducted in accordance with the protocol provided by Chiangnoon et al. [87]. ABTS 7.4 mM and potassium persulfate 2.6 mM were prepared by dissolving the reagents in deionized water. The ABTS radical cation (ABTS^•+^) was generated by mixing ABTS solution with potassium persulfate solution at room temperature in the dark for 24 h before use. The ABTS^•+^ solution was then diluted with absolute ethanol to achieve a 750 nm absorbance within the range of 0.7 ± 0.1. The reaction mixture consisted of varying concentrations: 3.91–2000 µg/mL of ascorbic acid, gallic acid, quercetin, and Trolox^®^, serving as a positive control [88], and different YTPS formulary and individual extract concentrations ranging from 9.77–5000 µg/mL (5 µL), including β-amyrin and stigmasterol in the range of 1.95–1000 µg/mL, followed by the addition of 145 µL of *ABTS*^•+^ solution. After a 15-min incubation period at room temperature in the dark, the absorbance of each reaction mixture was measured at 750 nm with a microplate reader. Linear regression analysis was established to determine the concentration of the YTPS formulary extract sample required to scavenge 50% of the ABTS radical (IC_50_).

$$ABTS\;radical\;scavenging\;activity\;(\%)=\frac{A-B}{A}\times 100\%$$

In this equation, A represents the absorbance observed in the ABTS reaction with the solvent control, while B represents the absorbance recorded when ABTS reacted with the extract.

##### Ferric Reducing Antioxidant Power (FRAP) Assay

The FRAP assay was carried out in accordance with the procedure described by Chittasupho et al. [88]. The FRAP reagent was composed of 10 mM TPTZ in 40 mM HCl, 20 mM FeCl_3_6H_2_O, and acetate buffer (300 mM, pH 3.6) in a ratio of 10:1:1 (*v*/*v*). A mixture of 180 µL of FRAP reagent and 20 µL of samples; YTPS formulary extract and individual extract ^®^ in the range of 9.77–5000 µg/mL; positive controls, namely, ascorbic acid, gallic acid, quercetin, and Trolox^®^ ranging between 3.91–2000 µg/mL; and, β-amyrin and stigmasterol in the range of 1.95–1000 µg/mL were thoroughly combined. After a 30-min incubation period at 37 °C, the absorbance at 570 nm was measured. A standard curve was established using a ferrous sulfate solution ranging from 9.8 to 2500 µM, and the results were expressed in terms of µmol Fe (II) equivalents.

#### 4.2.7. *In Vitro* Cytotoxicity

##### Cell Culture

The RAW 264.7 and HaCaT cells were grown in Dulbecco’s Modified Eagle Medium (DMEM) containing 10% fetal bovine serum (FBS), 2 mM L-glutamine, 100 units/mL penicillin, and 0.1 mg/mL streptomycin. The cells were cultured in a humidified incubator at 37 °C with 5% CO_2_. Regular checks were performed on the cells, and fresh media were provided as needed to sustain the logarithmic growth phase.

The THP-1 cells were grown in Roswell Park Memorial Institute (RPMI) 1640 Medium supplemented with 2 mM L-glutamine, 10% FBS, 50 units/mL penicillin, and 50 μg/mL streptomycin at 37 °C in a humidified atmosphere with 5% CO_2_ (CellXpert^®^, Eppendorf^®^, Eppendorf SE, Hamburg, Germany). Cells were periodically checked and media changed to maintain a logarithmic phase of growth.

The HEK293 cells were cultured in DMEM Nutrient Mixture F12-Ham (DMEM/F-12) (1:1) containing 2 mM L-glutamine with 10% FBS, 50 units/mL penicillin, and 50 μg/mL streptomycin at 37 °C in a humidified incubator with 5% CO_2_. Cell numbers were periodically assessed by counting under a microscope with a hemocytometer. Cell lines were subcultured with 0.25% Trypsin–EDTA for 5 min, and the enzyme reaction was inactivated by adding complete media.

##### Measurement of Cell Viability by MTT Assay

An MTT assay was conducted as per Chittasupho et al. [89]. The HaCaT and RAW 264.7 cells were trypsinized and collected from the flask. Cells were plated in 96-well plates at a density of 1 × 10^4^ cell/well. Cells were exposed to YTPS formulary extract diluted in 1% DMSO in serum-free medium ranging from 3.91 to 500 and 1.95 to 250 µg/mL for HaCaT and RAW 264.7 cells, respectively. The plates were incubated with 5% CO_2_ at 37 °C in a CO_2_ incubator for 24 h, followed by removing the media and adding 100 µL of DMEM with 10% FBS containing 0.5 mg/mL of MTT solution. After 2 h of incubation, the crystallized formazan was dissolved in 100% DMSO. The plates were read at 550 nm with a multi-mode microplate reader (Spectra MaxM3, Molecular Devices, Sunnyvale, CA, USA). Cell viability was evaluated using the equation below.

$$Cell\;viability\;\left(\%\right)=\frac{{Abs550}_{treated\;cells}}{{Abs550}_{untreated\;cells}}\times 100\%$$

##### Measurement of Cell Viability by AlamarBlue Assay

The AlamarBlue assay was carried out as stated by Bibič et al. [90]. After being removed from the flask, HEK293 and THP-1 cells were resuspended in DMEM/F-12 and RPMI1640 media, respectively. The cells were seeded in 96-well plates at a density of 1 × 10^4^ cells/well in a 200 µL volume. Prior to treatment, the HEK293 cell line was cultured for 24 h at 37 °C in a humidified environment with 5% CO_2_.After cell seeding, β-amyrin, stigmasterol, YTPS formulary extract, and individual extracts were diluted in different solvents. For an additional 24 h, the plates were kept in a humidified CO_2_ incubator. Following the incubation period, each well was added with 20 µL of the Resazurin dye solution (0.1 mg/mL) and placed into a CO_2_ incubator with 5% CO_2_ at 37 °C for 4 h. Using a Flexstation^®^ 3 multi-mode microplate reader (excitation at 570 nm and emission at 600 nm), the fluorescence of each 96-well plate was measured. Cell viability was calculated by subtracting background fluorescence from control cells and expressing it as a percentage.

$$Cell\;viability\;\left(\%\right)=\frac{{Abs}_{treated\;samples}}{{Abs}_{cell\;alone}}\times 100$$

#### 4.2.8. Anti-Inflammation Assay

##### Nitric Oxide Assay

Nitric oxide (NO) secretion was evaluated on the RAW 264.7 macrophages in accordance with Chittasupho et al. [89]. After plating cells in 96-well plates at a density of 1 × 10^4^ cells/well, the plates were incubated for 24 h. The cells were pretreated without or with lipopolysaccharide (LPS; 50 ng/mL) for 1 h and then exposed to β-amyrin (4–570 µM), stigmasterol (2–243 µM), and YTPS formulary extract (1.95–250 µg/mL) for 18 h. The collected supernatant was treated with Grise reagent and measured with a multi-mode microplate reader at 540 nm. The nitrile level was calculated against the nitric oxide standard regression curve.

##### Measurement of Cytokine Secretion by ELISA Assay

An ELISA assay was used to determine the secreted amounts of TNF-α, IL-6, and IL-1β according to the manufacturer’s instructions. Briefly, β-amyrin (20 µM), stigmasterol (20 µM), YTPS formulary extract (500–1000 µg/mL), individual plant extracts (50–100 µg/mL), and BAY 11-7085 (5 mM) were added into 24-well plates containing THP-1 cells (1 × 10^6^ cells/well). Each well was stimulated with LPS (0.01 µg/mL for IL-1β and TNF-α and 1 µg/mL for IL-6). For 3 h, the plates were in an incubator set at 37 °C and 5% CO_2_. The cell suspension was centrifuged at 2000 rpm for 5 min. After the incubation, the sandwich ELISA was performed. In summary, a 96-well plate was coated overnight at room temperature with a capture antibody. Subsequently, the capture antibody was blocked using reagent diluent, and standards along with samples were introduced. Following that, a detection antibody and streptavidin–HRP were added before the substrate solution was applied, and a stop solution was applied in the final step. Absorbance readings were obtained at 450 nm and 570 nm using a multi-mode microplate reader. 

#### 4.2.9. Statistical Analysis

The data were analyzed using GraphPad Prism version 9.5.0 with one-way ANOVA and Dunnett’s multiple comparisons test (statistical significance denoted by * *p* < 0.05, ** *p* < 0.01, *** *p* < 0.001, and **** *p* < 0.0001). The data are shown as mean ± standard deviation (mean ± S.D.) values, which were calculated from the average of three independent experiments.

## 5. Conclusions

The YTPS ethanolic extract, a Thai national polyherbal formula containing 13 different herbal ingredients, was analyzed for major compounds found in the formulary, β-amyrin and stigmasterol. The extract, at a non-toxic dose, exhibited an *in vitro* anti-inflammatory property by inhibiting pro-inflammatory cytokines, including IL-1β, IL-6, and TNF-α. In addition, the formulary also significantly diminished NO production in a dose-dependent manner. Our findings suggest that YTPS ethanolic extract has potential as an alternative topical remedy for osteoarthritis and inflammatory disease, with no cytotoxicity against kidney cells. 

## Figures and Tables

**Table 1:** The total phenolic and total flavonoid contents.

Samples	Total Phenolic Contents (mg GAE/g)	Total Flavonoid Contents (mg QAE/g)
YTPS formulary extract	117.73 ± 8.55	3148.06 ± 2.05
*Putranjiva roxburghii* Wall. extract	123.64 ± 5.17	2172.84 ± 6.74
*Senna siamea* (Lam.) H.S. Irwin & Barneby extract	114.29 ± 3.91	2546.67 ± 2.05
*Baliospermum solanifolium* (Burm.) Suresh extract	120.06 ± 2.45	2667.56 ± 14.37
*Cymbopogon nardus* (L.) Rendle extract	122.31 ± 5.71	1615.13 ± 11.73
*Tamarindus indica* L. extract	127.28 ± 1.47	2496.22 ± 2.05
*Melia azedarach* L. extract	109.94 ± 7.96	1659.49 ± 10.56
*Boesenbergia rotunda* (L.) Mansf. extract	142.04 ± 7.84	1340.00 ± 1.76
*Allium sativum* L. extract	100.65 ± 5.23	855.61 ± 2.35
*Alpinia galanga* (L.) Willd. extract	126.01 ± 0.42	636.86 ± 5.57
*Piper nigrum* L. extract	115.44 ± 1.34	1391.31 ± 0.88
*Ferula assa-foetida* L. extract	87.15 ± 0.45	1904.31 ± 4.40
*Aloe vera* (L.) Burm.f. extract	134.94 ± 4.56	2083.10 ± 7.04
*Allium ascalonicum* L. extract	115.89 ± 2.11	171.43 ± 11.73

**Table 2:** The IC_50_ values of samples for antioxidant assays.

Treatment	IC_50_ Values of DPPH Assay (µg/mL)	IC_50_ Values of ABTS Assay (µg/mL)	FRAP Values (µM)
Ascorbic acid	8.16 ± 0.30	24.23 ± 2.02	4150.33 ± 6.84
Gallic acid	6.84 ± 0.18	9.77 ± 0.77	4185.50 ± 2.77
Quercetin	6.71 ± 0.35	26.01 ± 1.31	3883.53 ± 13.58
Trolox^®^	10.72 ± 0.21	32.22 ± 0.32	4081.54 ± 10.56
YTPS formulary extract	79.61 ± 0.99	271.40 ± 10.02	100.99 ± 8.11
*P. roxburghii*	36.99 ± 1.53	96.98 ± 3.38	1788.21 ± 2.63
*S. siamea*	222.90 ± 1.60	564.50 ± 8.60	878.97 ± 18.66
*B. solanifolium*	417.80 ± 7.77	3903.00 ± 128.00	328.97 ± 8.47
*C. nardus*	176.80 ± 3.99	6419.50 ± 645.59	809.25 ± 7.12
*T. indica*	39.64 ± 1.01	858.0 ± 13.00	1821.25 ± 2.26
*M. azedarach*	311.80 ± 10.48	1334 ± 15.01	652.19 ± 4.40
*B. rotunda*	353.0 ± 10.05	446.30 ± 8.76	444.87 ± 16.35
*A. sativum*	1438.0 ± 8.49	8667 ± 95.46	253.35 ± 14.73
*A. galanga*	232.30 ± 24.59	962.90 ± 14.98	647.28 ± 8.20
*P. nigrum*	324.10 ± 28.85	1611 ± 15.28	774.16 ± 9.03
*A. vera*	226.90 ± 16.74	214.70 ± 6.01	457.65 ± 7.74
*A. ascalonicum*	1024.0 ± 8.72	2225 ± 77.93	353.86 ± 10.24

**Table 3:** Pro-inflammatory cytokines released from THP-1 cells after treatment.

Treatment	Concentration	IL-1β (pg/mL)	IL-6 (pg/mL)	TNF-α (pg/mL)
Control	-	0.00	0.00	0.00
LPS		22.51 ± 1.03	69.71 ± 1.06	64.96 ± 0.51
LPS + BAY 11-7085		0.00	0.00	0.00
LPS + β-amyrin	20 µM	8.65 ± 0.42 ****	57.57 ± 2.29 ****	11.69 ± 1.64 ****
LPS + stigmasterol	20 µM	11.56 ± 0.47 ****	64.54 ± 1.75	15.39 ± 0.72 ****
LPS + YTPS formulary	1000 µg/mL	20.54 ± 1.12	2.31 ± 0.14 ****	57.32 ± 3.78 ****
LPS + *P. roxburghii*	100 µg/mL	2.77 ± 0.36 ****	2.17±0.86 ****	16.29 ± 0.74 ****
LPS + *S. siamea*	100 µg/mL	0.00 ****	0.00 ****	0.00 ****
LPS + *B. solanifolium*	100 µg/mL	98.73 ± 7.22 ****	0.00 ****	36.62 ± 1.36 ****
LPS + *C. nardus*	100 µg/mL	8.16 ± 0.57 ****	16.02 ± 0.37 ****	14.94 ± 1.80 ****
LPS + *T. indica*	50 µg/mL	0.00 ****	0.00 ****	0.00 ****
LPS + *M. azedarach*	100 µg/mL	0.00 ****	0.00 ****	0.00 ****
LPS + *B. rotunda*	100 µg/mL	19.77 ± 2.14	0.00 ****	0.00 ****
LPS + *A. sativum*	100 µg/mL	11.80 ± 0.92 ****	93.67 ± 9.70 ****	24.42 ± 1.42 ****
LPS + *A. galanga*	100 µg/mL	0.00 ****	6.79 ± 0.35 ****	2.29 ± 0.62 ****
LPS + *P. nigrum*	100 µg/mL	0.00 ****	0.00 ****	0.00 ****
LPS + *F. assa-foetida* + *A. vera*	100 µg/mL	8.40 ± 0.89 ****	34.21 ± 4.45 ****	15.23 ± 1.86 ****
LPS + *A. ascalonicum*	100 µg/mL	5.51 ± 0.33 ****	17.50 ± 3.14 ****	6.15 ± 1.05 ****

The data were analyzed by GraphPad Prism version 9.5.0 with one-way ANOVA and Dunnett’s multiple comparisons test (statistical significance denoted by **** *p* < 0.0001 compared with cells treated with LPS).

**Table 4:** YTPS herbal composition.

No.	Plant Scientific Name	Parts Used	%*w*/*w*	Voucher Specimen
1	*P. roxburghii*	Leaves	37	TTM0005479
2	*S. siamea*	Leaves	9	TTM0005489
3	*B. solanifolium*	Leaves	9	TTM0005473
4	*C. nardus*	Rhizomes and leaves	9	TTM0005477
5	*T. indica*	Leaves	9	TTM0005469
6	*M. azedarach*	Leaves	9	TTM0005488
7	*B. rotunda*	Rhizomes and roots	2	TTM0005476
8	*A. sativum*	Bulbs	2	TTM0005474
9	*A. galanga*	Rhizomes	2	TTM0005475
10	*P. nigrum*	Fruits	2	TTM0005490
11	*F. assa-foetida*	Resin from roots	2	TTM1000743
12	*A. vera*	Resin	2	TTM1000744
13	*A. ascalonicum*	Bulbs	2	TTM0005478

## Data Availability

Data are contained within the article and Supplementary Materials.

## Supplementary Materials

The following supporting information can be downloaded at: https://www.mdpi.com/article/10.3390/ph17081018/s1, Figure S1: Key results obtained from the identification and quantification of bioactive compounds and biological activities of YTPS extract; Figure S2; The standard calibration curve of (A) quercetin and (B) gallic acid; Figure S3; HPLC chromatogram of the YTPS formulary extract spiked with beta-amyrin and stigmasterol; Figure S4; The standard calibration curve for (A) β-amyrin and (B) stigmasterol using HPLC analysis; Figure S5; Standard curve of ferric reducing antioxidant power assay using ferrous sulfate Table S1: The IC_50_ values of samples after treatment with HEK293 cells.
