# Supplementary material for: Anti-Inflammatory Activities of Yataprasen Thai Traditional Formulary and Its Active Compounds, Beta-Amyrin and Stigmasterol, in RAW264.7 and THP-1 Cells
Source: Pharmaceuticals (Basel). 2024 Aug 1;17(8):1018. doi: 10.3390/ph17081018 (PMC11357128; doi:10.3390/ph17081018)
Supplement: Supplementary file 1 [file pharmaceuticals-17-01018-s001.zip › pharmaceuticals-3083647-supplementary.pdf]

# Anti-Inflammatory Activities of Yataprasen Thai Traditional Formulary and Its Active Compounds, Beta-Amyrin and Stigmasterol, in RAW264.7 and THP-1 Cells

**Jaenjira Angsusing [1,2], Sudarshan Singh [3,4], Weerasak Samee [5], Sarin Tadtong [6], Leanne Stokes [7], Maria O'Connell [7], Hanna Bielecka [7], Nopparut Toolmal [2], Supachoke Mangmool [8] and Chuda Chittasupho [9,*]**

[1] Ph.D. Degree Program in Pharmacy, Faculty of Pharmacy, Chiang Mai University, CMU Presidential Scholarship, Chiang Mai 50200, Thailand; jaenjira_ang@cmu.ac.th
[2] Thai Traditional Medicine Research Institute, Department of Thai Traditional and Alternative Medicine, Ministry of Public Health, Bangkok 10100, Thailand; nopparut.toolmal@gmail.com
[3] Faculty of Pharmacy, Chiang Mai University, Chiang Mai 50200, Thailand; sudarshansingh83@hotmail.com
[4] Office of Research Administration, Chiang Mai University, Chiang Mai 50200, Thailand
[5] Department of Pharmaceutical Chemistry, Faculty of Pharmacy, Srinakharinwirot University, Nakhon Nayok 26120, Thailand; weerasak@g.swu.ac.th
[6] Department of Pharmacognosy, Faculty of Pharmacy, Srinakharinwirot University, Nakhon Nayok 26120, Thailand; sarin@g.swu.ac.th
[7] School of Pharmacy, University of East Anglia, Norwich, Norwich Research Park, Norfolk NR4 7TJ, UK; l.stokes@uea.ac.uk (L.S.); m.oconnell@uea.ac.uk (M.O.); h.bielecka@uea.ac.uk (H.B.)
[8] Department of Pharmaceutical Care, Faculty of Pharmacy, Chiang Mai University, Chiang Mai 50200, Thailand; supachoke.man@cmu.ac.th
[9] Department of Pharmaceutical Sciences, Faculty of Pharmacy, Chiang Mai University, Chiang Mai 50200, Thailand
* Correspondence: chuda.c@cmu.ac.th; Tel.: +66-5394-4342; Fax: +66-5394-4390

**Figure S1.** Key results obtained from the identification and quantification of bioactive compounds and biological activities of YTPS extract.

**Figure S2.** The standard calibration curve of (A) quercetin and (B) gallic acid.

**Figure S3.** HPLC chromatogram of the YTPS formulary extract spiked with beta-amyrin and stigmasterol.

**Figure S4.** The standard calibration curve for (A) β-amyrin and (B) stigmasterol using HPLC analysis.

**Figure S5.** Standard curve of ferric reducing antioxidant power assay using ferrous sulfate.

**Table S1.** The IC50 values of samples after treatment with HEK293 cells.

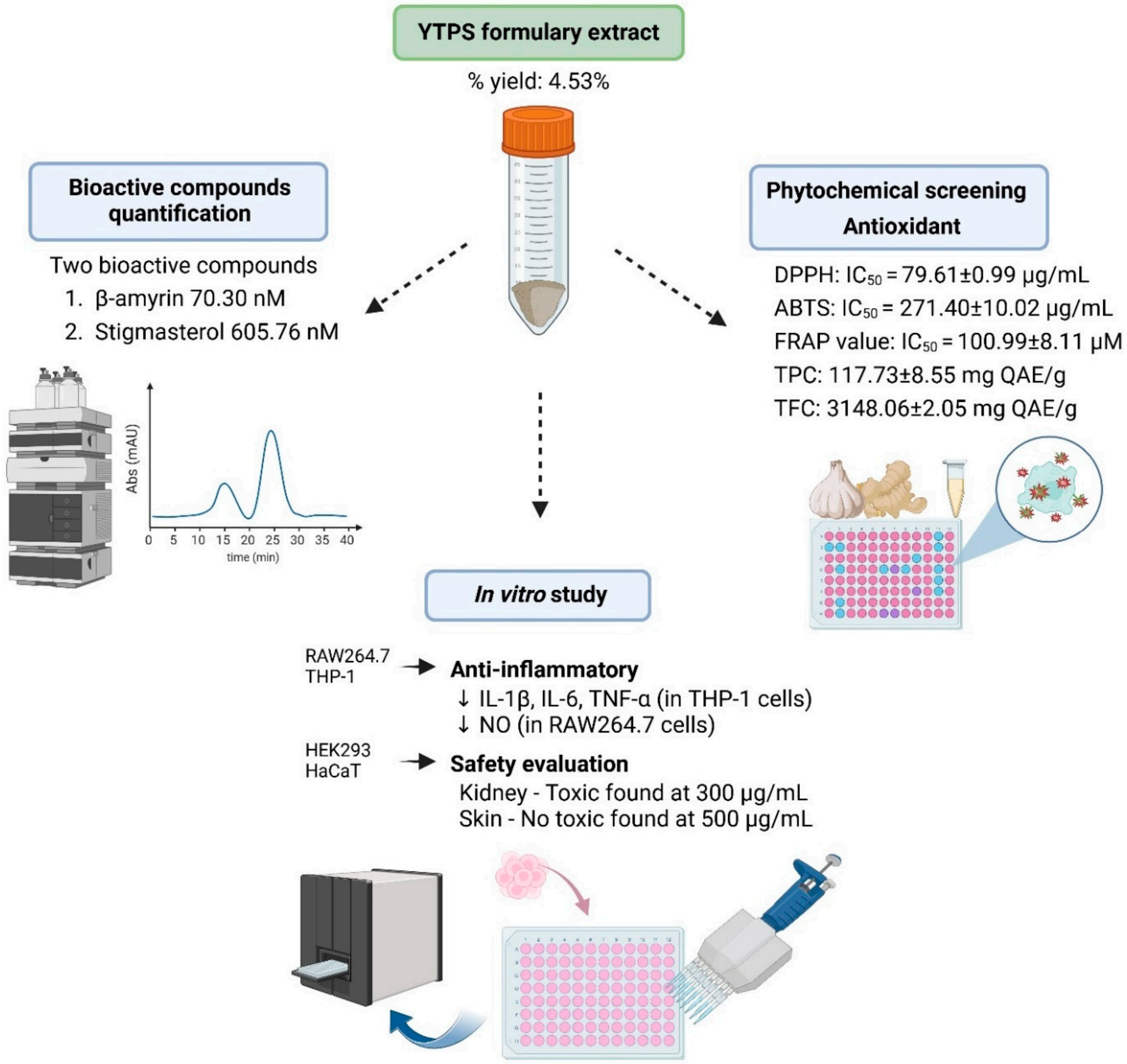


**Figure S1.**

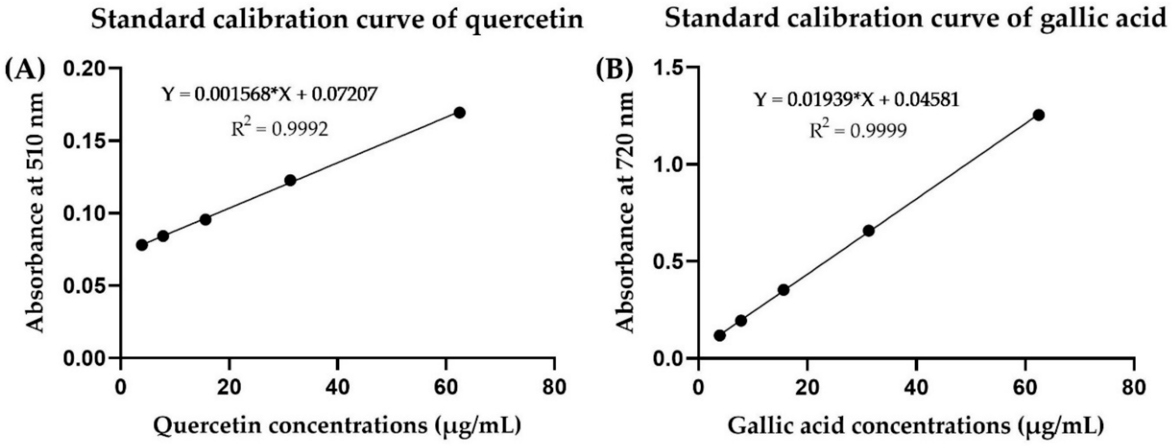


**Figure S2.**

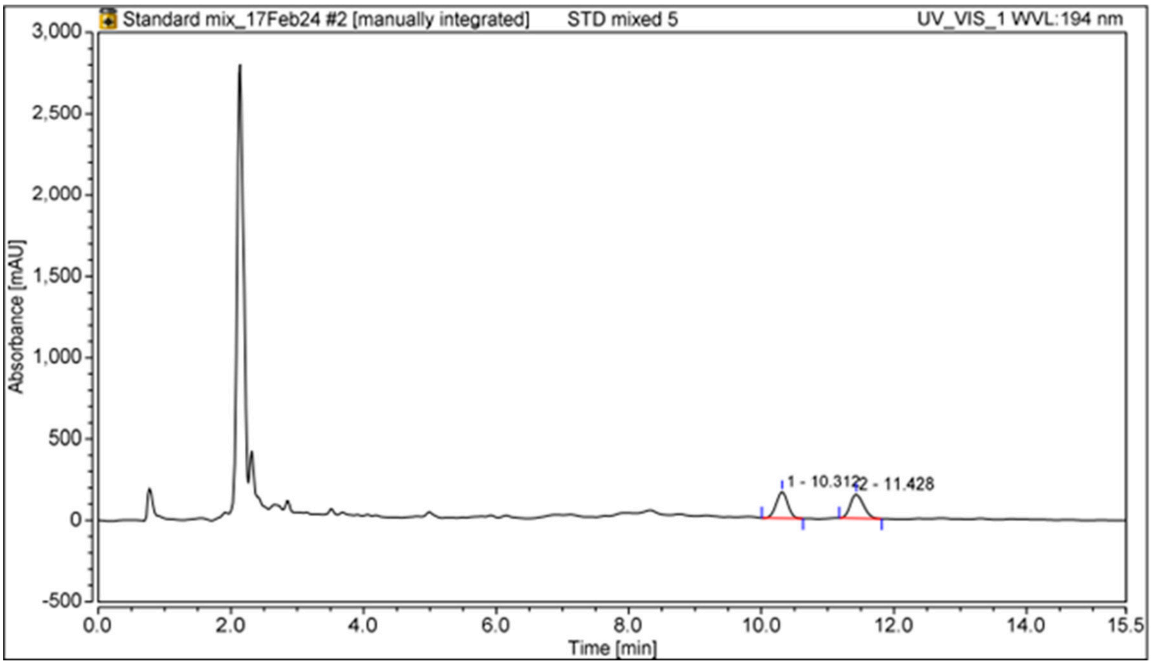


**Figure S3.**

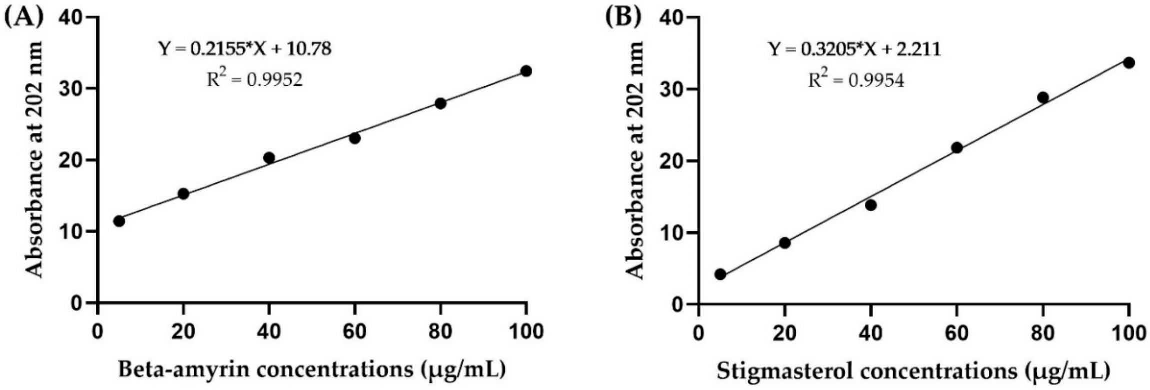


**Figure S4.**

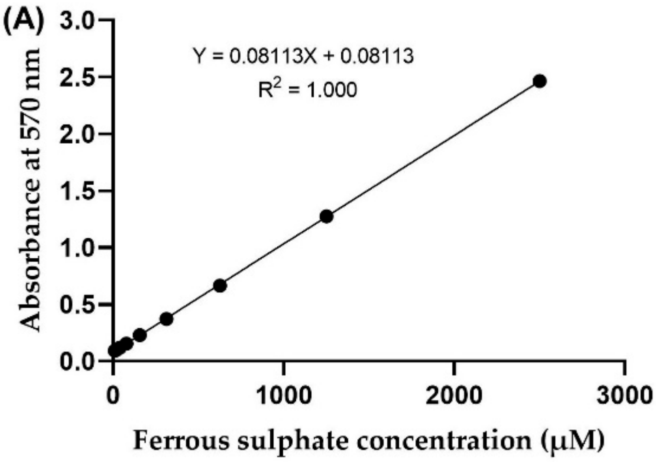


**Figure S5.**

**Table S1.**

| Treatment | $IC_{50}$ value |
|---|---|
| YTPS formulary extract | 326.2 μg/mL |
| β-amyrin | 28.85 μM |
| Stigmasterol | 2.92 μM |
| *Putranjiva roxburghii* | 6.01 μg/mL |
| *Senna siamea* | 6.37 μg/mL |
| *Baliospermum solanifolium* | 2.91 μg/mL |
| *Cymbopogon nardus* | 0.80 μg/mL |
| *Tamarindus indica* | 1.65 μg/mL |
| *Melia azedarach* | 1.67 μg/mL |
| *Boesenbergia rotunda* | 6.06 μg/mL |
| *Allium sativum* | 1.01 μg/mL |
| *Alpinia galanga* | 1.45 μg/mL |
| *Piper nigrum* | 7.99 μg/mL |
| *Allium ascalonicum* | 9.55 μg/mL |
